# Characterization of the novel mitochondrial genome segregation factor TAP110 in *Trypanosoma brucei*

**DOI:** 10.1242/jcs.254300

**Published:** 2021-03-08

**Authors:** Simona Amodeo, Ana Kalichava, Albert Fradera-Sola, Eloïse Bertiaux-Lequoy, Paul Guichard, Falk Butter, Torsten Ochsenreiter

**Affiliations:** 1 Institute of Cell Biology, University of Bern, 3012 Bern, Switzerland; 2 Graduate School for Cellular and Biomedical Sciences, University of Bern, 3012 Bern, Switzerland; 3 Institute of Molecular Biology, 55128 Mainz, Germany; 4 Department of Cell Biology, University of Geneva, Sciences III, 1211 Geneva, Switzerland

**Keywords:** Kinetoplast DNA, kDNA, Mitochondrial genome segregation machinery, Tripartite attachment complex, TAC, *Trypanosoma brucei*, Ultrastructure expansion microscopy, U-ExM

## Abstract

Proper mitochondrial genome inheritance is important for eukaryotic cell survival. *Trypanosoma brucei*, a protozoan parasite, contains a singular mitochondrial genome, the kinetoplast (k)DNA. The kDNA is anchored to the basal body via the tripartite attachment complex (TAC) to ensure proper segregation. Several components of the TAC have been described; however, the connection of the TAC to the kDNA remains elusive. Here, we characterize the TAC-associated protein TAP110. We find that both depletion and overexpression of TAP110 leads to a delay in the separation of the replicated kDNA networks. Proteome analysis after TAP110 overexpression identified several kDNA-associated proteins that changed in abundance, including a TEX-like protein that dually localizes to the nucleus and the kDNA, potentially linking replication and segregation in the two compartments. The assembly of TAP110 into the TAC region seems to require the TAC but not the kDNA itself; however, once TAP110 has been assembled, it also interacts with the kDNA. Finally, we use ultrastructure expansion microscopy in trypanosomes for the first time, and reveal the precise position of TAP110 between TAC102 and the kDNA, showcasing the potential of this approach.

This article has an associated First Person interview with the first author of the paper.

## Abstract (precis)

**Summary:** TAP110 is a novel mitochondrial genome segregation factor in *Trypanosoma brucei* that associates with the previously described TAC component TAC102. Ultrastructure expansion microscopy reveals its proximal position to the kDNA.

## INTRODUCTION

Mitochondrial organelles are a defining feature of eukaryotic cells. They perform a large number of different functions ranging from catabolic reactions, like oxidative phosphorylation (Friedman and Nunnari, 2014), to anabolic processes, like iron-sulfur cluster assembly (Braymer and Lill, 2017), and Ca^2+^ homeostasis (Giorgi et al., 2018). The vast majority of the mitochondrial proteins are encoded and expressed from the nuclear genome, while only a small set of proteins, mostly belonging to respiratory chain complexes, are encoded on the genome of the organelle. In *Trypanosoma brucei,* a parasitic protist, the mitochondrial genome is organized in a complex structure named kinetoplast DNA (kDNA). It consists of ∼25 large (23 kbp) circular DNA molecules that encode 16 genes of the oxidative phosphorylation chain, two ribosomal proteins and two ribosomal RNAs (Ramrath et al., 2018; Shapiro and Englund, 1995). Twelve of the mitochondrial genes require post-transcriptional modifications by RNA editing prior to translation on the mitochondrial ribosomes (Hajduk and Ochsenreiter, 2010; Read et al., 2016; Simpson, 2003; Stuart et al., 2005). The guide RNAs involved in this process are encoded on minicircles (1 kbp), of which about 5000 are catenated into the kDNA network forming a disc like structure (Cooper et al., 2019). In that network, the minicircles are oriented perpendicularly to the horizontal plane of the disc (Chen et al., 1995; Delain and Riou, 1969; Diao et al., 2015; Rauch et al., 1993). The maxicircles are interwoven into the minicircle network and also interlocked with each other (Shapiro, 1993; Shapiro and Englund, 1995). Replication of the kDNA occurs during G1 of the parasite cell cycle, just prior to the start of nuclear DNA replication. Our current model of kDNA replication predicts that, for replication initiation, the minicircles are released into the kinetoflagellar zone (KFZ) (Bruhn et al., 2010; Drew and Englund, 2001; Hines and Ray, 2011; Hoeijmakers and Weijers, 1980; Jensen and Englund, 2012; Klingbeil et al., 2002; Milman et al., 2007). The replication products are subsequently separated and transported by an unknown mechanism to the opposing ends of the kDNA disc, where they are further processed and eventually reattached to the network (Jensen and Englund, 2012; Povelones, 2014). Once all minicircles have been replicated, the daughter networks are segregated through the movement of the basal bodies of the flagellum (Robinson and Gull, 1991). The physical connection between the kDNA and the basal bodies that mediates segregation has been described in electron microscopy studies and termed the tripartite attachment complex (TAC) (Ogbadoyi, 2003). The TAC consists of (1) the exclusion zone filaments, a region between the basal bodies and the outer mitochondrial membrane that is devoid of ribosomes, (2) the differentiated mitochondrial membranes and (3) the unilateral filaments that connect the inner mitochondrial membrane to the kDNA (Ogbadoyi, 2003). Several proteins of this structure have been characterized and the analysis of their common features have provided us with an operational definition of a TAC component (Povelones, 2014; Schneider and Ochsenreiter, 2018). TAC proteins are (1) localized between the basal body and the kDNA in whole cells, as well as in isolated flagella; (2) depletion of a TAC protein leads to kDNA missegregation and eventually kDNA loss; (3) TAC proteins are dispensable in the γL262P bloodstream form *T. brucei* cell line that is capable of normal cell growth with and without a mitochondrial genome due to a compensatory mutation in the γ subunit of the ATP synthase (Dean et al., 2013).

Of all currently analyzed TAC components, TAC102 is the TAC protein that is the most proximal to the kDNA. However, it remains unclear whether TAC102 binds directly to kDNA disc or whether other proteins are mediating this process (Hoffmann et al., 2016; Trikin et al., 2016). The closest interactor of TAC102 is the transmembrane domain containing protein p166, which is localized at the inner mitochondrial membrane (Baudouin et al., 2020; Zhao et al., 2008). Three outer mitochondrial membrane components of the TAC (TAC40, TAC42 and TAC60; Käser et al., 2017; Schnarwiler et al., 2014) as well as two components in the exclusion zone filaments (p197 and TAC65; Hoffmann et al., 2018; Käser et al., 2017; Zhou et al., 2010) are also essential for proper kDNA segregation. Furthermore, there are a number of proteins including TbTBCCD1, pATOM36, α-KDE2, AEP1 and polymerase IC (Pol IC) that are in or associated with the TAC and have additional functions in the cell (André et al., 2013; Käser et al., 2016; Miller et al., 2020; Ochsenreiter et al., 2008; Sykes and Hajduk, 2013). Experimental evidence from the mitochondrial polymerase Pol IC and the minicircle replication factor MiRF172 support the idea of a physical interaction between the replication machinery and the TAC (Amodeo et al., 2018; Miller et al., 2020). During G1 of the trypanosome cell cycle, the TAC is assembled *de novo* in a hierarchical process from the maturing basal body towards the kDNA (Hoffmann et al., 2018; Schneider and Ochsenreiter, 2018). While we and others have identified components of all three TAC regions, it remains unknown how and through which components the TAC is connected to the kDNA. In order to identify novel components of the TAC that might interact with the kDNA, we used an N-terminally tagged TAC102 protein as bait to purify interacting partners. Here, we present that the novel TAC-associated protein TAP110 interferes with kDNA segregation and, based on its position in ultrastructure expansion microscopy (U-ExM), might be part of the structure connecting the TAC to the kDNA.

## RESULTS

TAP110 (Tb927.11.7590) is a basic (pI=8), 110 kDa, hypothetical conserved protein with a predicted mitochondrial targeting sequence at the N-terminus but otherwise no detectable domains or similarities to proteins outside the Kinetoplastea in the public databases (Fig. S1A). We identified TAP110 and the hypothetical protein Tb927.11.6660 in biochemical approaches as the two most abundant interaction partners of TAC102 (Fig. S1B,C). TAP110 contains six post-translational modifications in the form of methylated arginine residues (Fig. S1A) (Fisk et al., 2013). A phylogenetic analysis shows that TAC102 and TAP110 share a common evolutionary history, and, similar to other TAC components, TAP110 is not found in Perkinsela (Fig. S1D, also see Discussion).

### TAP110 localization

**Fig. 1.**
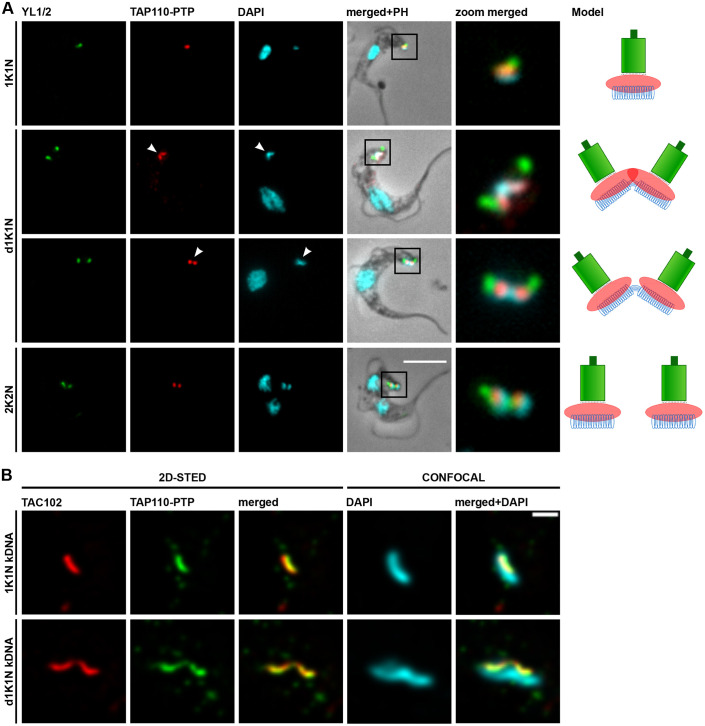
**Localization of TAP110 in *T. brucei* BSF cells.** (A) Representative immunofluorescence microscopy images of TAP110–PTP-expressing BSF cells during different stages of the cell cycle (1K1N, dK1N, 2K2N). The mature basal bodies (green) were detected with the YL1/2 monoclonal antibody. TAP110–PTP (red) was detected by means of the anti-Protein A antibody. The kDNA and the nucleus were stained with DAPI (cyan). The panel on the right side shows a simplified model of TAP110 localization during the cell cycle. Green depicts the basal bodies, red TAP110 and blue the kDNA. Scale bars: 5 μm. The zoom merged panel is shown at 4× magnification relative to the main panels. Arrowheads point towards duplicated TAP110 signals and duplicating or duplicated kDNAs. (B) Deconvoluted 2D-STED immunofluorescence images of TAP110–PTP (green)- and TAC102 (red)-stained BSF cells. TAC102 (red) was detected with the anti-TAC102 monoclonal antibody, TAP110-PTP (green) and the kDNA (cyan) were detected as described above. The TAC102 and TAP110 signals were acquired by 2D-STED, and the kDNA by confocal microscopy. Scale bar: 500 nm. d, duplicating or duplicated; K, kDNA; N, nucleus; PH, phase contrast.

To localize the TAP110 protein, we tagged it *in situ* at the C-terminus with a PTP epitope tag in New York single marker (NYsm) bloodstream form (BSF) *T. brucei* (Schimanski et al., 2005; Wirtz et al., 1999). We performed immunofluorescence microscopy and used an anti-Protein A antibody to detect TAP110–PTP. Based on colocalization studies with the basal body marker YL1/2 and the DNA stain DAPI, the protein localizes between the kDNA and the basal bodies (Fig. 1A). TAP110 localization during the cell cycle resembled the typical localization pattern of a TAC component. Two signals for TAP110 are discernable before the kDNA is segregated, but only after the separation and maturation of the daughter basal body (Fig. 1A, arrowheads).

Based on the proximity to TAC102 and the kDNA in epifluorescence microscopy, we decided to compare TAP110 and TAC102 localization by super-resolution microscopy using stimulated emission depletion (STED) microscopy. TAP110 and TAC102 colocalized with a Pearson correlation coefficient of 0.841 with a minimum *x-y* resolution of 37.9 nm (*n*=16) (Fig. 1B).

### Depletion of TAP110 by RNAi

**Fig. 2.**
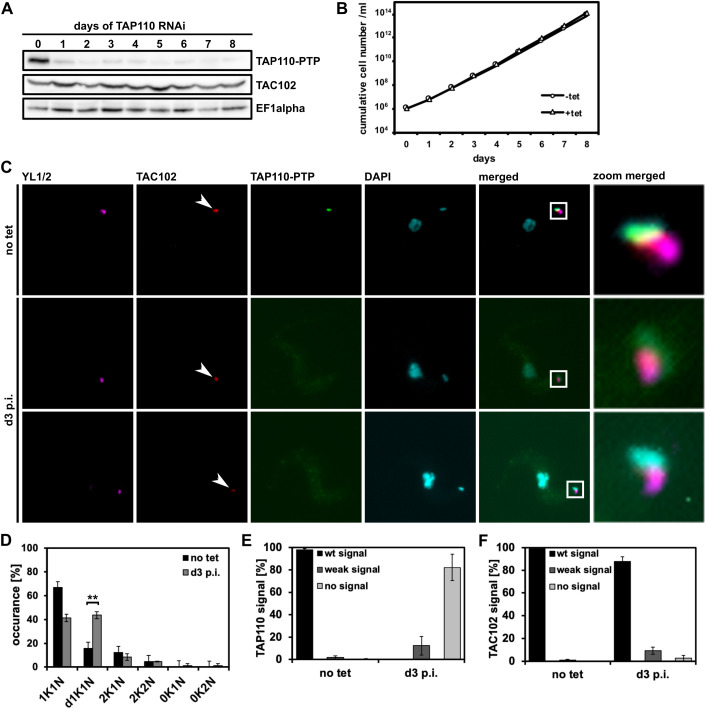
**Depletion of TAP110 mRNA by RNAi in BSF cells.** (A) Western blot of whole-cell lysates showing depletion of TAP110–PTP protein at different days of the RNAi induction. TAP110–PTP was detected by use of anti-rabbit IgGs and TAC102 was detected using a monoclonal anti-TAC102 antibody. EF1α serves as a loading control (*n*=1). (B) Growth curve of tet-inducible BSF TAP110 RNAi TAP110-PTP cells (*n*=1). (C) Immunofluorescence images of non-induced cells (no tet) and cells at day 3 post induction (d3 p.i.). The signals are represented by maximum intensity projections from image stacks. Basal bodies, TAP110-PTP, TAC102 and DNA were detected as described in Fig. 1. Scale bar: 5 μm. The zoom merged panel is shown at 7× magnification relative to the main panels. (D) Quantification of the relative occurrence of kDNA networks and nuclei in non-induced cells (no tet) and cells at day 3 post-induction (d3 p.i.). Results are mean±s.d. for ≥100 cells for each condition and replicate (*n*=3). (E) Quantification of TAP110 signal from the experiment shown in C. A Results are mean±s.d. for ≥100 cells for each condition and replicate (*n*=3). (F) Quantification of TAC102 signal as performed in E. K, kDNA; N, nucleus; PH, phase contrast. **P≤0.01 (two-tailed unpaired *t*-test).

To study the function of TAP110, we depleted the mRNA by RNAi using a tetracycline (tet) inducible RNAi vector (Bochud-Allemann and Schneider, 2002) in NYsm BSF cells that contained an endogenously PTP-tagged allele of TAP110, as described above. The knockdown was efficient but not complete, as monitored by probing for the PTP-tagged TAP110 protein in western blots (Fig. 2A). Although these cells did not display a growth defect (Fig. 2B), we observed an increase of duplicated non-segregated kinetoplasts (d1K1N) from 16% in non-induced cells to 44% on day 3 post TAP110 depletion (*P*<0.01, Fig. 2D; 31% on day 2, Fig. S5D). In the same cell line, the mean network size was increased by 15% (*P*<0.001, Welch *t*-test, Wilcoxon test, and permutation test) at 6 days after RNAi induction (Fig. S2). To test whether TAP110 depletion had an effect on TAC assembly, we probed for TAC102 using a monoclonal antibody in TAP110-depleted cells and observed a loss of TAC102 signal in 3% of the population at 3 days post RNAi induction in immunofluorescence microscopy images (Fig. 2F). Furthermore, 9% of the induced cells showed a weaker signal for TAC102 at this timepoint (Fig. 2C, lowest panel; Fig. 2F).

In conclusion, the depletion of TAP110 leads to an increase of replicating and replicated non-segregated kDNA networks in the population, and has a minor effect on the localization of TAC102, a TAC component of the unilateral filaments (ULFs; Fig. 2D). For the quantification analyses, we used ≥100 cells for each time point and replicate (*n*=3).

### Overexpression of TAP110

**Fig. 3.**
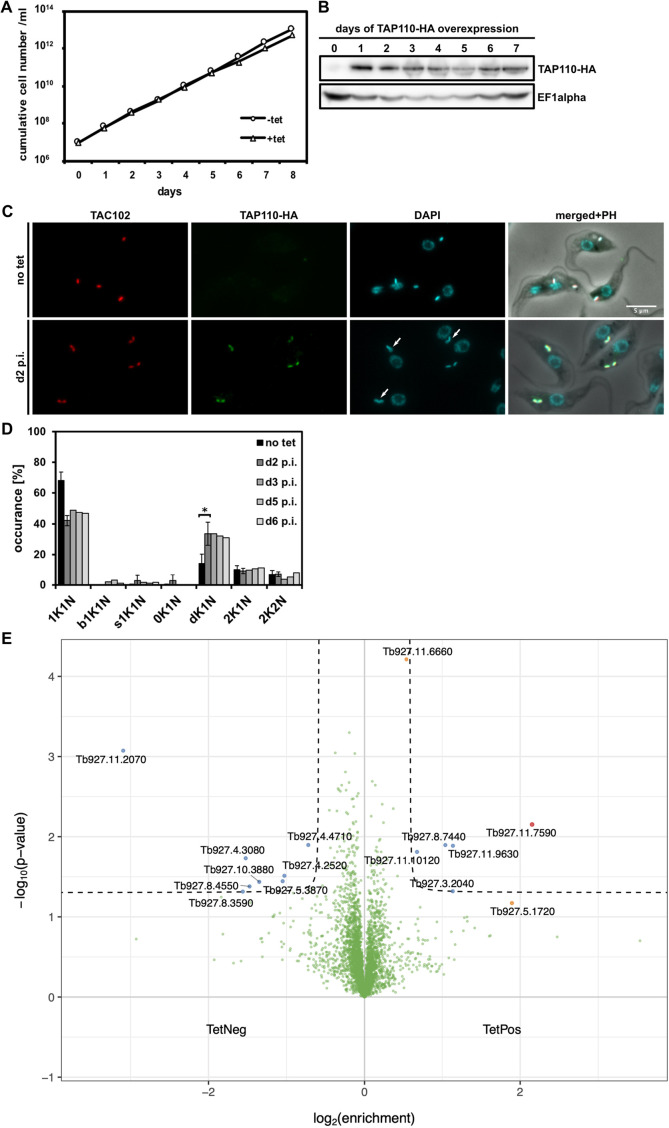
**Overexpression of TAP110-HA in PCF cells.** (A) Growth curve of tet-inducible PCF TAP110–HA cells (*n*=1). (B) Western blot of whole cell lysates showing expression of TAP110-HA protein at different days of overexpression. TAP110-HA was detected by an anti-HA antibody; EF1α serves as a loading control (*n*=1). (C) Immunofluorescence microscopy images of non-induced (no tet) and induced cells at day 2 post induction of the overexpression construct (d2 p.i.). TAC102 and DNA were detected as described in Fig. 1 and TAP110-HA was detected by an anti-HA antibody. Scale bar: 5 μm. Arrows point towards duplicating or duplicated non-segregated kDNAs. (D) Quantification of the relative occurrence of kDNA networks and nuclei in cells before inducing the overexpression (no tet) and at different days post induction (d2–d6 p.i.). Results are mean±s.d. for ≥100 cells for each condition, day [and replicate (*n*=3) in case of no tet and d2 p.i.]. (E) Volcano plot of proteins in tet positive (d2 p.i.) against tet negative cells. Highlighted in red is TAP110 (enrichment 4.45); the proteins highlighted in blue are possible interactors passing the threshold of a *P*<0.05 and log_2_ fold change >1 or <−1 (dashed lines). Further highlighted in orange are possible interactors not passing the threshold of *P*<0.05 or log_2_ fold change >1 or <−1. bK, big kDNA; dK, duplicating kDNA; K, kDNA; N, nucleus; PH, phase contrast; sK, small kDNA. *P<0.05 (two-tailed unpaired *t*-test).

To further evaluate the function of TAP110, we created a tetracycline inducible overexpression cell line with a HA-tagged ectopic version of TAP110 in 29-13 procyclic form (PCF) cells (generation of bloodstream form clones overexpressing TAP110 was unsuccessful). Overexpression for 8 days did not lead to a growth defect, but on day 2 post induction of overexpression (Fig. 3A,B), we observed an increase of cells with replicated, non-segregated kDNA networks (d1K1N) from 14% in non-induced cells to 34% [*P*<0.05; a number of ≥100 cells for each condition and replicate (*n*=3) was analyzed; Fig. 3C,D], similar to what was seen for depletion of TAP110 in BSF cells (Fig. 2D). We also analyzed the overall proteome changes upon TAP110-HA overexpression using mass spectrometry. For this, we induced expression of TAP110–HA for 2 days and then compared the total cell proteome to non-induced cells. Aside from TAP110, which was enriched 4.5-fold, we detected six other proteins with increased abundance, while eight were decreased (Fig. 3E; Table S1). Among the total of 15 proteins that were changed in abundance, eight are predicted mitochondrial proteins, and of those, five had a basic isoelectric point (including TAP110), typical for proteins of the inner ULFs (Aslett et al., 2010; Claros and Vincens, 1996; Gluenz et al., 2007; Peikert et al., 2017; Zhang et al., 2010). Two of the proteins, including Tb927.11.6660, a TEX-like protein, are localized to the kDNA as shown by a high-throughput localization screen (TrypTag; Dean et al., 2017). As a reminder, Tb927.11.6660 was also detected as a putative TAC102 interactor in the TAC102 immunoprecipitation (Fig. S1). We depleted Tb927.11.6660 by RNAi and observed that it was not essential for the survival of the parasite (data not shown). We also tagged Tb927.11.6660 at its C-terminus with a PTP tag and observed that it was present at the kDNA throughout the cell cycle and in the nucleus during nuclear S phase (Fig. S3). Of the other seven proteins that changed in abundance, one is a putative kinesin localized to a structure at the axoneme, the hook complex, two proteins show an endocytic localization pattern, and two were found to be localized to the flagellum, as shown in a previous high-throughput localization screen (Aslett et al., 2010; Dean et al., 2017).

Since the cell growth was not affected by the depletion (Fig. 2B), and overexpression of TAP110 only had a minor effect on cell growth (Fig. 3A), we tested two other overexpression clones which showed the same growth behavior and also increase of d1K1N cells. Interestingly one clone even showed a slight kDNA loss phenotype (Fig. S4). Furthermore, we tested whether the addition of a stress stimulus would reveal a phenotype in TAP110-depleted and overexpressing cells. For this we applied heat stress (33°C) in PCF cells (TAP110 overexpression), and treated BSF (TAP110 RNAi) cells with ethidium bromide, a DNA-intercalating dye. The addition of ethidium bromide to the medium led to a strong growth retardation phenotype starting on day 3 post addition of the compound. However, there was no difference between the wild-type and TAP110-depleted cells (Fig. S5A–D). In the heat-stress condition, we observed a slightly stronger growth retardation in induced cells grown at 33°C (compare Fig. 3A and Fig. S5E). When we analyzed DAPI-stained, heat-stressed PCF cells at day 8 post induction of TAP110 overexpression, we observed an increased number of cells with abnormal kDNA network content (Fig. S5F). We analyzed ≥150 cells for each condition and detected an increase in the proportion of cells with small (7%) and large (6%) kDNAs. Furthermore, 28% of the population had replicated non-segregated kDNA networks (d1K1N) and 6% of the 2K1N cells had missegregated kDNAs (u2K1N) (Fig. S5G).

### Impact of TAC102 depletion on TAP110

**Fig. 4.**
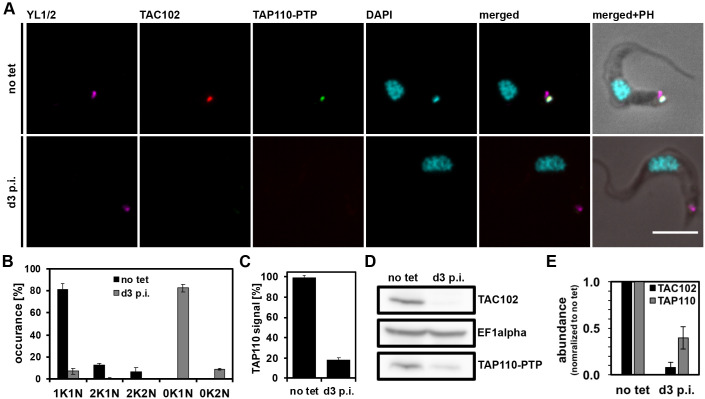
**Effect of TAC102 depletion on TAP110 localization and abundance.** (A) Immunofluorescence microscopy images of non-induced (no tet) and induced cells at day 3 post induction (d3 p.i.). Images were produced as described in Fig. 1. Scale bar: 5 μm. (B) Quantification of the relative occurrence of kDNA networks and nuclei before inducing the RNAi (no tet) and at day 3 post induction (d3 p.i.) from the imagery shown in A. Results are mean±s.d. for ≥100 cells for each condition (i.e. no tet, d3 p.i.) and biological replicates (i.e. different clones, *n*=3). (C) Quantification of TAP110 signal from the experiment shown in A. Results are mean±s.d. for ≥100 cells for each condition and biological replicate (*n*=3). (D) Western blot showing depletion of TAC102 and TAP110–PTP at day 3 of RNAi (d3 p.i.). Probing for EF1αserves as a loading control. (E) Quantification of TAP110 and TAC102 signal intensities from western blots as seen in D. Signals were normalized to EF1α to allow comparison and then to no tet values to calculate residual signal at day 3 post induction (mean±s.d., *n*=3, signals from three different clones serve as biological replicates). K, kDNA; N, nucleus; PH, phase contrast.

To further analyze potential interactions of TAP110 with the TAC, we investigated the effect of TAC102 RNAi on TAP110. Previous studies have shown that the TAC is assembled hierarchically from the base of the flagellum to the kDNA (Hoffmann et al., 2018). Consequently, depletion of a TAC protein distal to the kDNA leads to loss of the kDNA-proximal TAC components (Hoffmann et al., 2018). Thus, if TAP110 is closer to the kDNA than TAC102, depletion of the latter would be expected to lead to a loss of TAP110. We performed immunofluorescence microscopy on cells from day 3 post TAC102 depletion (Fig. 4A). We analyzed ≥100 cells for each condition and biological replicate to confirm the previously described TAC102 depletion phenotype (*n*=3) (Trikin et al., 2016) (Fig. 4B). We also observed that 85% of the TAC102-depleted cells had no signal for TAP110 (Fig. 4C). We further performed western blot analysis of whole-cell lysates and probed for TAC102 and TAP110, and found that both proteins were significantly depleted (TAC102 to 8%, TAP110 to 40%, *n*=3; Fig. 4D,E). Thus, the presence of TAC102 is required for proper localization of the majority of TAP110.

### Ultrastructure expansion microscopy

Based on the TAC102 RNAi experiments, we predicted TAP110 to be proximal to the kDNA. However, STED super-resolution microscopy largely showed colocalization of the two proteins (see Fig. 1B). In order to further improve the resolution, we established ultrastructure expansion microscopy (U-ExM) for insect form *T. brucei* cells.

**Fig. 5.**
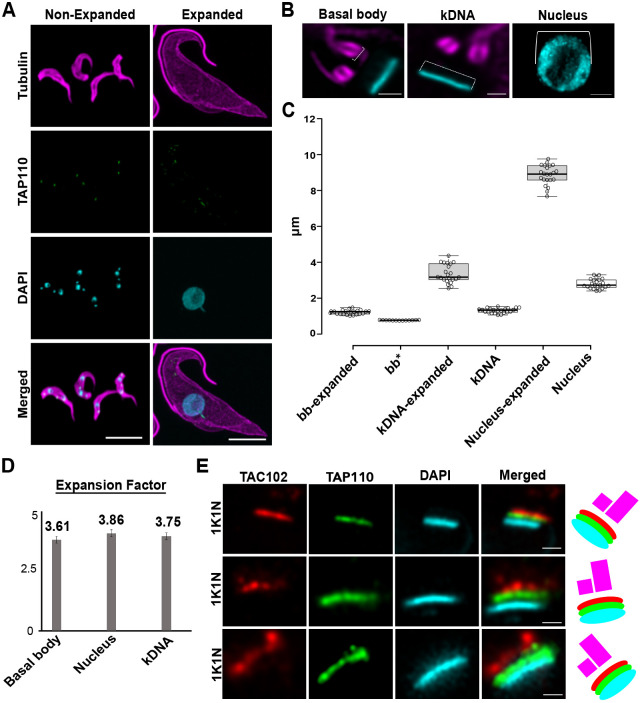
***T. brucei* expansion with U-ExM.** (A) Non-expanded and expanded PCF cells stained with α-tubulin (magenta; Alexa Fluor 594), TAP110 (green; Oregon Green 488), kDNA (cyan; DAPI) and imaged by confocal microscopy followed by deconvolution. Scale bars: 20 μm. (B) Magnified views of the basal body, kDNA and nucleus. Scale bars: 2.5 μm. (C) Measurements of basal body size, kDNA length and nucleus diameter in non-expanded and expanded cells [see brackets in B, expanded basal bodies, kDNAs and nuclei for 22 cells each; non-expanded kDNAs and nuclei each 22 cells each; * corresponds to non-expanded basal body measurements from TEM (*n*=12 cells)]. The box represents the 25–75th percentiles, and the median is indicated. The whiskers extend to data points less than 1.5× the interquartile range (Tukey). (D) Expansion factor calculated as the ratio between non-expanded and expanded basal body, kDNA and nucleus from measurements obtained in C. Results are mean±s.d. (E) Representative images of localized TAP110 (green; Oregon Green 488), TAC102 (red, Alexa Fluor 594) and kDNA (cyan, DAPI) in expanded cells. A diagram of the localization of the basal bodies (magenta), TAC102 (red), TAP110 (green) and the kDNA (cyan) is shown on the right. Scale bars: 1 μm.

Immunostaining with an anti-tubulin antibody in combination with confocal microscopy showed that the PCF trypanosome cells were ‘equally’ expanded in all three dimensions, largely retaining the trypomastigote morphology of an elongated cell body that tapers at the anterior and posterior end (Fig. 5A; Fig. S6). To further investigate the expansion process inside the cell, we stained the nucleus and kDNA with DAPI and compared non-expanded with expanded cells. We observed isotropic expansion of the nucleus by a factor of 3.86±0.594 (mean±s.d., *n*=22; Fig. 5B–D), while the expansion of the kDNA disc was largely limited to the planar axis of the disc, increasing the diameter by a factor of 3.75±0.628 (*n*=22; Fig. 5B–D). As a third parameter, we measured the expansion of the basal body diameter and compared it to those found in thin section electron microscopy imagery (*n*=12) from non-expanded chemically fixed cells. We found the basal body to be isotropically expanded by a factor of 3.61±0.14 (*n*=22, Fig. 5B–D; Fig. S6; potential shrinkage of thin section electron microscopy imagery was not compensated for).

We next explored whether U-ExM could increase resolution in the region of the TAC close to the kDNA. We immunostained the cells using a monoclonal antibody for TAC102 and anti-HA antibody for TAP110, and stained the DNA with DAPI. The extra staining seen for TAP110 in the overview image of the expanded cell (Fig. 5A), is non-specific staining that occurs with some antibodies at the intersection between the cell and the slide, and is visualized owing to the maximum intensity projection. Confocal microscopy focusing on the TAC in the expanded cells showed that TAP110 is closer to the kDNA than TAC102 (Fig. 5E).

### kDNA and TAP110 localization

**Fig. 6.**
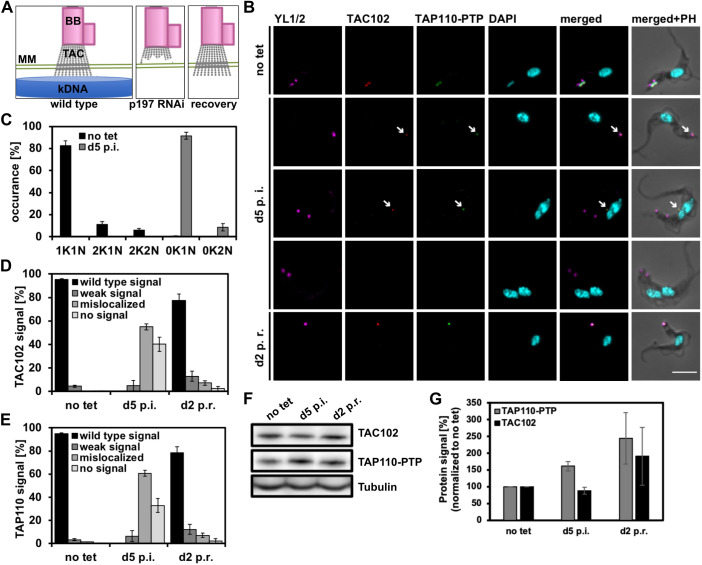
**Recovery of TAP110 in γL262P p197 RNAi TAP110-PTP BSF cells.** (A) Diagram showing how depletion of p197 by RNAi in γL262P cells leads to loss of the TAC and kDNA; when RNAi against p197 is released (recovery) in the same cells, the TAC reassembles. (B) Immunofluorescence microscopy of the γL262P p197 RNAi recovery experiment. Non-induced (no tet), induced cells at day 5 post induction (d5 p.i.) and cells at day 2 post recovery (d2 p.r.) are shown. Scale bar: 5 μm. Arrows point towards weak and mislocalized TAC102 and TAP110 signals. (C) Quantification of the relative occurrence of kDNA networks and nuclei from experiment shown in B. Results are mean±s.d. for ≥150 cells per time point. (D) Quantification of TAC102 signals from the experiment shown in B [mean±s.d. for ≥150 cells for each time point and replicate (*n*=3)]. (E) Quantification of TAP110 signals from the experiment shown in B [mean±s.d. for ≥150 cells for each time point and replicate (*n*=3)]. (F,G) Representative western blot and the corresponding quantification (mean±s.d.) from three independent experiments showing TAC102 and TAP110-PTP in non-induced cells (no tet), cells at day 5 of p197 RNAi (d5 p.i.) and cells at day 2 after removal of tetracycline (d2 p.r.). Tubulin serves as loading control. BB, basal body; K, kDNA; N, nucleus, PH, phase contrast.

In order to test whether the kDNA is required for proper localization of TAP110, we created a dyskinetoplastic cell line through depletion of the TAC component p197 in γL262P BSF cells, which are able to survive without kDNA (Dean et al., 2013). As previously shown, re-expression of p197 in the dyskinetoplastic population leads to *de novo* assembly of the TAC without any kDNA (see simplified model in Fig. 6A; Hoffmann et al., 2018). We monitored the localization of endogenously tagged TAP110 in these cells. At 5 days post p197 RNAi depletion, 100% of the cells were dyskinetoplastic. Through immunofluorescence microscopy, we observed that 55% of the cells had TAC102 and 61% had TAP110 mislocalized in the mitochondrion. Interestingly, in almost all cases the mislocalized TAC102 and TAP110 signals were colocalized. Furthermore, we observed a loss of TAC102 in 40% of the cells and loss of TAP110 in 33% of the cells, while 5% had a reduced signal for TAC102 and 6% for TAP110 (Fig. 6B,C). After re-expression of p197 (RNAi released) we found that TAC102 and TAP110 signals returned to wild-type localization and intensity (Fig. 6B). For quantification of the experiment, we analyzed the DAPI, TAC102 and TAP110 signals for ≥150 cells for each time point and replicate (*n*=3) (Fig. 6C–E). We observed that TAC102 and TAP110 behave the same in the course of the recovery experiment (Fig. 6D,E). We also controlled for the abundance of the proteins by western blotting and found that, despite the loss of localization, both TAC102 and TAP110 were still present in the cell (Fig. 6F,G).

### TAP110 complex

**Fig. 7.**
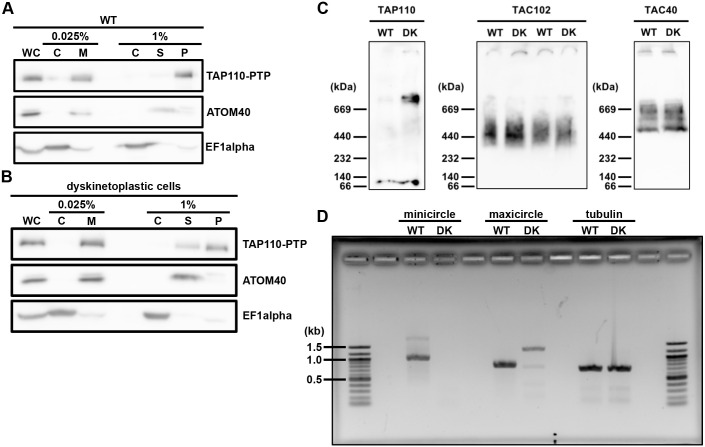
**Biochemical analysis of TAP110 by western blot and blue native PAGE.** (A) Western blot of different digitonin extraction fractions obtained from wild-type (WT) γL252P p197 RNAi TAP110–PTP BSF cells. ATOM40, mitochondrial marker; EF1α, cytosolic marker. (B) Western blot of different digitonin extraction fractions obtained from dyskinetoplastic (DK) γL252P p197 RNAi TAP110-PTP BSF cells. (C) Blue Native PAGE from wild-type and dyskinetoplastic cells lysed and soluble mitochondrial fractions. TAP110–PTP, TAC102 and EF1α were detected as described in Fig. 2. TAC40–HA was detected with an anti-HA antibody, and ATOM40 with an anti-ATOM40 antibody. On the blot probed for TAC102, we loaded WT and DK from both cell lines, the TAP110–PTP (left) and the TAC40–HA (right). (D) PCR products using WT or dyskinetoplastic cells (DK) gDNA and mini-, maxicircle or tubulin primers. C, cytosolic fraction; M, mitochondrial fraction; P, insoluble mitochondrial fraction; S, soluble mitochondrial fraction; WC, whole cells. All experiments shown are representative of at least three replicates.

The mitochondrial protein TAC102 and other TAC components can be partially solubilized by digitonin extractions as shown in earlier studies (Hoffmann et al., 2018; Käser et al., 2017; Trikin et al., 2016). At a concentration of 0.025% digitonin, cytoplasmic components can be separated from crude organellar structures containing the mitochondrial organelle. This crude mitochondrial pellet can then be lysed with 1% digitonin to solubilize mitochondrial proteins and also partially TAC proteins. We performed this extraction with the uninduced γL262P p197 RNAi cell line, which still contained the mitochondrial genome, and with the dyskinetoplastic version of this cell line. In the kDNA-containing cells, TAP110 was not soluble (Fig. 7A), whereas in the dyskinetoplastic cells, TAP110 could be partially solubilized (Fig. 7B). We then performed Blue Native PAGE with extracts from wild-type and dyskinetoplastic cells (Fig. 7C). As previously shown, the TAC components TAC102 and TAC40 form complexes of 440 kDa and 500–750 kDa, respectively (Hoffmann et al., 2018). TAP110 was detected in a complex of ∼669 kDa in the dyskinetoplastic cell line. To verify dyskinetoplasticity of the cells, we performed PCR on DNA extracted from wild-type and dyskinetoplastic cells (Fig. 7D). Based on the PCR, we believe that the dyskinetoplastic cells indeed are kDNA free, or at least minicircle free. Thus, TAP110 forms a large complex that is partially soluble in dyskinetoplastic cells and only partially overlaps with the complex size previously described for the outer membrane component TAC40.

### TAP110 association with flagella

**Fig. 8.**
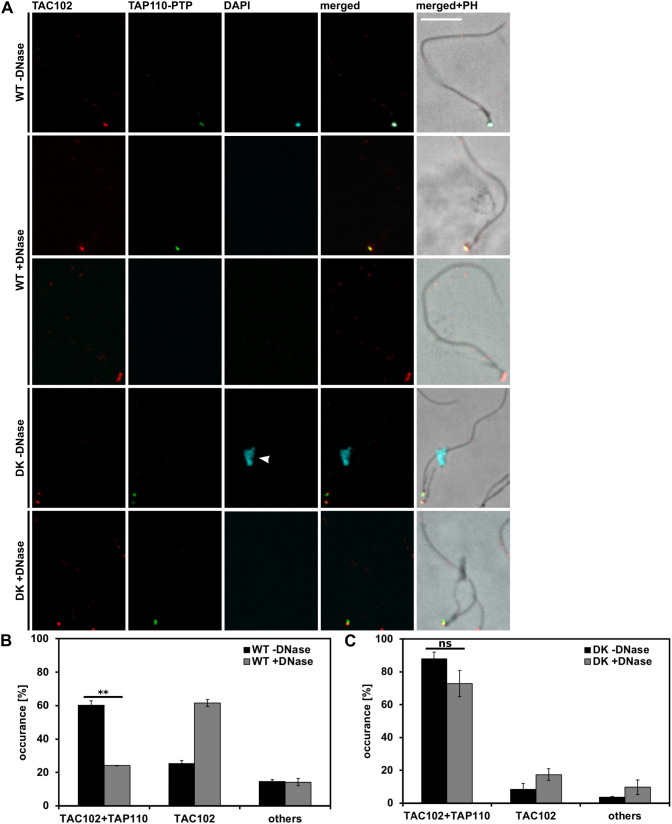
**Localization of TAP110-PTP in flagellar extracts of wild-type and dyskinetoplastic γL262P p197 RNAi TAP110–PTP BSF cells.** (A) Immunofluorescence microscopy images of extracted flagella either without DNaseI (−DNase) or with DNaseI treatment (+DNase). Staining was performed as described in Fig. 1. Scale bar: 5 μm. Arrowhead points towards nuclear DNA. (B) Quantification (mean±s.d.) of wild-type (WT) flagella of the experiment shown in A. For the first replicate we analyzed ≥145 flagella for each condition (WT, with and without DNase; DK with and without DNase). For replicates two and three, ≥60 flagella were analyzed for each condition (WT, with and without DNase; DK with and without DNase). Flagella with TAC102 and TAP110–PTP signals (TAC102+TAP110), flagella with TAC102 signal only (TAC102) and other flagella (others) were counted. (C) Quantification (mean±s.d.) of dyskinetoplastic (DK) flagella of experiment shown in A. Quantification was performed as described in B. Others, describes mainly flagella with no signal, rarely with a signal for TAP110 only. **P≤0.01; ns, not significant (two-tailed unpaired *t*-test).

The TAC complex is largely insoluble, and TAC proteins remain associated with the flagellum after extraction from the cell (Schneider and Ochsenreiter, 2018). To test whether this is true for TAP110, we isolated flagella by detergent extraction with Triton-X 100 as described previously (Dolan et al., 1986; Ogbadoyi, 2003; Trikin et al., 2016). To furthermore test what effect the mitochondrial DNA has on the association of TAP110 to the TAC we (1) treated some samples with DNaseI during the extraction and (2) used dyskinetoplastic cells that lack the kDNA *in vivo*. The isolated flagella were analyzed for TAC102 and TAP110 by immunofluorescence microscopy (Fig. 8A). In 60% of the flagella we detected TAC102 and TAP110 together, while in 25% only TAC102 was present (Fig. 8A,B). When we treated the flagella with DNaseI, the fraction of TAC102-containing flagella remained unchanged, while TAP110 was then only detected in 24% of the flagella (Fig. 8A,B) indicating that removing the kDNA during extraction leads to an increased solubility of TAP110. In 88% of the flagella isolated from dyskinetoplastic cells, TAC102 and TAP110 were co-detected. The presence of TAP110 in these flagella slightly decreased upon treatment with DNaseI, suggesting that either the dyskinetoplastic cells still contained some DNA to which TAP110 associated or the buffer/enzyme combination itself increased the solubility of TAP110 (Fig. 8A,C). Nonetheless, this increased solubility of TAP110 was not significant for dyskinetoplastic cells, while it was significant in wild-type cells (compare Fig. 8B and 8C).

## DISCUSSION

Based on a comparative phylogenetic analysis TAC102 and TAP110 seem to be inherited together (Fig. S1D) and, similar to what is seen for other TAC components, TAP110 is not found in Perkinsela, an endosymbiotic kinetoplastid without basal body and flagellum, that consequently also misses a TAC. Interestingly, TAC102 and TAP110 are also absent from the *Bodo saltans* genome, while all other TAC orthologs can be found in this free-living kinetoplastid. This might suggest, that while most of the TAC machinery is conserved in all Kinetoplastea, the components in proximity to the DNA have adapted to the different kDNA conformations or replication mechanisms.

We previously described a number of criteria for proteins of the TAC, one of which is the localization between the kDNA disc and the basal body of the flagellum (Schneider and Ochsenreiter, 2018). To determine the precise localization of TAP110, we used STED super-resolution microscopy and found it to be colocalized with TAC102 in the unilateral filament region inside the mitochondrion. In an attempt to further increase the resolution, we established ultrastructure expansion microscopy (U-ExM) for insect form trypanosomes, and evaluated the expanded cells by confocal immunofluorescence microscopy. In general, the typical morphology of a trypomastigote cell was retained during the expansion process (Fig. 5) and we were able to elucidate structures such as the subpellicular microtubule array or the microtubule quartet at the basal body, as well as the nine-fold symmetry of triplet microtubules of the barrel shaped pro-basal body (Fig. S6E). The quasi-isotropic expansion was also confirmed by the analysis of nucleus and basal body shape (Fig. S6). Interestingly, *in situ*, the kDNA only expanded in the horizontal plane of the disc, while the height of the disc remained largely unchanged. The kDNA is mainly composed of catenated minicircles that are relaxed and predicted to be oriented perpendicularly to the horizontal plane of the disc (Chen et al., 1995; Delain and Riou, 1969; Diao et al., 2015; Rauch et al., 1993). The height of the kDNA disc is determined by the size of each minicircle (1 kb) and reaches its maximum when the minicircles are completely stretched out, which is apparently already the case in the unexpanded cells, and therefore does not allow for further expansion. The diameter of the kDNA disc and its expansion, however, depend on the packaging of the minicircles in the horizontal plane. Tight packaging, as predicted in the model, would thus likely allow for relaxation and expansion, as we detected in our experiments. After we evaluated the use of U-ExM for *T. brucei*, we applied it to elucidate the localization of TAP110 relative to TAC102 and the kDNA. So, while STED super-resolution microscopy suggested that TAC102 and TAP110 colocalize, U-ExM revealed that TAP110 is the kDNA-proximal protein, which is in good agreement with our current hierarchical model of TAC assembly and the TAC102 and TAP110 RNAi data (Figs 2 and 4). In summary, we established U-ExM in trypanosomes. This improved resolution by a factor of three or four while maintaining overall structural features of the cell and allowed us to determine orientation of TAP110 and TAC102 relative to the kDNA; two proteins that otherwise seemed to colocalize.

Aside from their localization between the basal body and the kDNA, previously characterized TAC components also remain associated with flagella during isolation, supporting the model of a structural protein complex with a stability similar to that of the axoneme. For TAC102, this is irrespective of the presence or absence of kDNA. For TAP110 the situation is more complex. If we remove the kDNA from the flagella during extraction through DNaseI treatment, the number of flagella that contain detectable levels of TAC102 and TAP110 decreases by more than half (Fig. 8B), suggesting that a fraction of TAP110 requires the kDNA or other kDNA-connected proteins to be stably associated with the TAC. Interestingly, this requirement is decreased in flagella isolated from cells that do not contain any kDNA to begin with (dyskinetoplastic cells, Fig. 8C). In these cells, TAP110 still colocalizes with TAC102 and remains with the TAC throughout the extraction. This suggests that TAP110 does not require kDNA for its proper localization in the TAC region, which is similar to TAC102 and the other known TAC components (Hoffmann et al., 2018).

The association of TAP110 with the kDNA is also evident from its biochemical behavior during solubilization of the TAC complex. While TAC102 becomes partially soluble irrespective of the presence or absence of kDNA, TAP110 is completely insoluble as long as kDNA is present (Fig. 7; Hoffmann et al., 2018; Trikin et al., 2016). We have also tried to express TAP110 in *E. coli* to perform DNA-binding assays with the purified protein, but have been unsuccessful in producing soluble protein.

Aside from their specific localization, TAC proteins are functionally characterized as segregation factors. Thus, depletion of any of the TAC components leads to a characteristic missegregation and eventually a kDNA-loss phenotype (Hoffmann et al., 2018; Schneider and Ochsenreiter, 2018; Trikin et al., 2016). Although the depletion of TAP110 leads to changes in kDNA content (Fig. 3; Fig. S2, Fig. S5), the cells do not display kDNA loss or missegregation phenotypes, otherwise typical for TAC components. This might be explained by the presence of other proteins with redundant function or the incomplete depletion of TAP110. Interestingly, overexpression of TAP110 leads to a very similar phenotype to its depletion. There are several possible explanations. The overexpression of a tagged version of TAP110 might have a dominant-negative effect and thus display a similar phenotype as the knockdown. Alternatively, segregation of the TAC and kDNA might depend on the accurate number of TAP110 molecules and thus provide a means of integrating the TAC and kDNA segregation with the cell cycle (see below).

To test whether and how TAP110 overexpression might influence other proteins associated with TAP110, we quantified the proteome during the overexpression of TAP110 (Fig. 3). Consistent with the phenotype, the overexpression of TAP110 only led to minor changes in the total cell proteome and half of these proteins are annotated as mitochondrial or kDNA-associated proteins. Among the list of proteins that changed in expression level (Table S1), one was a kDNA linked protein, Tb927.11.6660, which was previously identified in a screen for TAC102 interactors (Fig. S1). Tb927.11.6660 has strong similarities to TEX proteins, which are highly conserved bacterial proteins that likely function in a variety of transcriptional processes. Tb927.11.6660 localizes at the kDNA and the nucleus, which is consistent with its predicted mitochondrial, as well as nuclear, localization sequences (Fig. S3). While the nuclear localization is predominantly observed during nuclear S-phase, the protein seems to be at the kDNA during the entire cell cycle. It is tempting to speculate that TAP110 and Tb927.11.6660 might provide a link for the coordinated replication and segregation of the mitochondrial and nuclear genome. We hypothesize that the increase in proteins with endocytic localization patterns might be involved in degradation of increased amounts of TAP110 through lysosomal proteolysis. Furthermore, we attribute the changing levels of certain flagellar proteins as being caused by the increased number of d1K1N cells in the population. This shift in cell cycle profile will likely change the relative abundance of certain flagellar proteins due to the formation of the new flagellum and flagellar pocket during this cell cycle stage. In summary, the overexpression seems to impact only very few proteins, several of which have a direct link to the kDNA and one potentially providing a link to mitochondrial signaling.

We have described a novel kDNA segregation factor that might be involved in the TAC and, with Tb927.11.6660, provide a potential link to mitochondrial–nuclear communication, a model that can be tested in future experiments.

## MATERIALS AND METHODS

### *T. brucei* cell culture conditions

Procyclic form (PCF, 29-13) *T. brucei* cells were cultured in semi-defined medium-79 (SDM-79, custom made by Life Technologies; Brun and Schönenberger, 1979) supplemented with 10% fetal calf serum (FCS), 15 μg/ml geneticin and 25 μg/ml hygromycin at 27°C. Bloodstream form (BSF) *T. brucei* New York single marker (NYsm) cells (Wirtz et al., 1999) and γL262P BSF cells (Dean et al., 2013) were cultured at 37°C and 5% CO_2_ in Hirumi-modified Iscove's medium 9 (HMI-9, based on IMDM from GIBCO, 12440; Hirumi and Hirumi, 1989) supplemented with 10% FCS containing 2.5 μg/ml geneticin for NYsm, and with 2.5 μg/ml geneticin and 0.5 μg/ml puromycin for γL262P.

### Transfections of *T. brucei* cells

For transfections, we dissolved 10 μg of linearized plasmid or PCR product in 100 μl transfection buffer (90 mM sodium phosphate pH 7.3, 5 mM KCl, 0.15 mM CaCl_2_, 50 mM HEPES pH 7.3) (Burkard et al., 2007). For BSF and PCF transfection, we used 4×10^7^ and 10^8^ mid-log phase cells, respectively. Cells were resuspended in 100 μl transfection buffer containing the DNA, transferred into Amaxa Nucleofector cuvettes and transfections were conducted in the Amaxa Nucleofector II using program Z-001 (panel V 1.2 kV, panel T 2.5 kV, panel R 186 Ohm, panel C 25 μF) for BSF and program X-014 for PCF.

Then, we recovered the transfected cells for 20 h in medium without antibiotics. After recovery, we selected for correct integration of the construct with appropriate antibiotics (5 μg/ml blasticidin, 2.5 μg/ml geneticin, 2.5 μg/ml hygromycin, 2.5 μg/ml phleomycin or 0.5 μg/ml puromycin for BSF cells, and 10 μg/ml blasticidin or 1 μg/ml puromycin for PCF cells). Expression of the RNAi and overexpression constructs was induced by addition of tetracycline (tet) to a final concentration of 1 μg/ml for BSF and PCF cells.

### DNA constructs

TAP110–PTP was created by amplification of the TAP110 open reading frame (ORF) (Tb927.11.7590) positions 2242 to 2922 from genomic NYsm DNA and was cloned between the ApaI and EagI (NEB) sites of the pLEW100 based PTP tagging vector (Schimanski et al., 2005). We linearized the resulting plasmid with XcmI (NEB) prior to transfection. TAP110 RNAi targeting the ORF (positions 2081 to 2629) was cloned into a tet-inducible RNAi vector (Bochud-Allemann and Schneider, 2002) in two steps by cloning with the restriction enzymes BamHI HF, HindIII HF, XbaI and XhoI (NEB) to generate the later hairpin loop double-stranded RNA (dsRNA) for RNAi. The final plasmid was linearized with NotI HF (NEB) prior to transfection. The ORF of TAP110 was amplified and inserted without the stop codon by cloning with the restriction enzymes HindIII HF and XhoI (NEB) into a modified pLew100 vector for overexpression (Wenger et al., 2017; Wirtz et al., 1999).

For the Tb927.11.6660-PTP construct, the ORF positions 2397 to 2805 were amplified as described above and cloned between the ApaI and EagI sites. We used SnaBI (NEB) to linearize the plasmid prior to transfection.

### Standard immunofluorescence analysis

To analyze the localization of TAP110, TAC102 and basal body proteins, we used immunofluorescence analysis as described previously (Amodeo et al., 2018). Primary and secondary antibodies were diluted as follows: polyclonal rabbit-anti-Protein A (cat. no. P3375, Sigma) detecting the PTP epitope, 1:2000; rat YL1/2 antibody detecting tyrosinated tubulin as present in the basal body (Kilmartin et al., 1982), 1:100,000; monoclonal mouse TAC102 antibody (Trikin et al., 2016), 1:2000; and rabbit-anti-HA (cat. no. H6908, Sigma), 1:1000; Alexa Fluor^®^ 488 goat anti-rabbit IgG (H+L) (cat. no. A27034, Invitrogen); Alexa Fluor^®^ 594 goat anti-mouse IgG (H+L) (cat. no. R37121, Molecular Probes by Life Technologies); Alexa Fluor^®^ 647 goat anti-rat IgG (H+L) (cat. no. A-21247, Life Technologies), all 1:1000. We acquired the images with the Leica DM5500 B microscope (Leica Microsystems) and the 100× oil immersion phase contrast objective. Then, we used the LAS X software (Leica Microsystems) and ImageJ to analyze the images.

### Super-resolution 2D stimulated emission depletion microscopy

PTP-tagged TAP110 BSF cells were used to analyze the TAP110 and TAC102 localization in more detail with stimulated emission depletion (STED) microscopy. Cells were spread on no. 1.5 cover glasses (Marienfeld), fixed, permeabilized and mounted as described previously (Hoffmann et al., 2018). Polyclonal rabbit anti-Protein A antibody (Sigma) and monoclonal mouse-anti-TAC102 antibody were used as described above. The Alexa Fluor^®^ 594 goat anti-rabbit IgG (H+L) (cat. no. A-11012, Invitrogen) and the Atto 647N goat anti-mouse IgG (H+L) (cat. no. 50185, Sigma) were used 1:500 in 4% BSA and incubated for 1 h. To compare the localization of the two proteins we used the SP8 STED microscope (Leica, with a 100× oil immersion objective and the LAS X Leica software). Images were acquired as *z*-stacks with a *z*-step size of 120 nm and a *x-y* resolution of 37.9 nm. For the TAP110–PTP and the TAC102 signal, the 594 nm and 647 nm excitation laser and the 770 nm depletion laser were used. Owing to non-availability of suitable depletion laser, the DAPI signal was acquired with confocal settings. We deconvoluted the images with the Huygens professional software.

### Calculation of Pearson correlation coefficient of TAP110–PTP and TAC102 signals obtained from 2D STED microscopy

To analyze the colocalization of TAC102 and TAP110 we calculated Pearson's R value. For this, we first selected the kDNA region as a region of interest and created a two-color channel image, then we applied the coloc 2 plugin of the ImageJ software according to the manufacturer's user guide (Rueden et al., 2017; Schindelin et al., 2017). This was performed for 30 different selected kDNA regions for the TAC102 and TAP110 signal.

### SDS-PAGE and western blotting

To analyze presence and/or abundance of a protein of interest in whole-cell lysates and fractions from digitonin and flagellar extraction, we used western blot analysis. Samples were prepared as described previously (Amodeo et al., 2018). Approximately 5×10^6^ cells or cell equivalents were loaded per lane of a SDS-PAGE gel. The proteins were separated by electrophoresis and then transferred onto a PVDF membrane. Blocking and detection of proteins of interest (PTP- and HA-tagged proteins, EF1αand ATOM40) was performed as described previously (Amodeo et al., 2018). α-tubulin was detected by the monoclonal mouse anti-α-tubulin antibody (1:20,000, cat. no. T5168, Sigma) and the rabbit anti-mouse-IgG conjugated to horseradish peroxidase (1:10,000, Dako).

### Mass spectrometry and data analysis

Protein lysates from induced (day 2) and non-induced TAP110 overexpressing whole cells were separated on 10% NOVEX gradient SDS gel (Thermo Scientific) for 8 min at 180 V in 1× MES buffer (Thermo Scientific). Proteins were fixated and stained with a Coomassie solution [0.25% Coomassie Blue G-250 (Biozym), 10% acetic acid and 43% ethanol]. The gel lane was cut into slices, minced, and destained with a 50% ethanol and 50 mM ammonium bicarbonate pH 8.0 solution. Proteins were reduced in 10 mM DTT for 1 h at 56°C and then alkylated with 50 mM iodoacetamide for 45 min at room temperature in the dark. Proteins were digested with trypsin (Sigma-Aldrich) overnight at 37°C. Peptides were extracted from the gel using a mixture of acetonitrile (30%) and 50 mM ammonium bicarbonate pH 8.0 solution twice, and three times with pure acetonitrile, which was subsequently evaporated in a concentrator (Eppendorf) and loaded on an activated C18 material (Empore) StageTip (Rappsilber et al., 2007).

For mass spectrometric analysis, peptides were separated on a 50 cm self-packed column (New Objective) with 75 µm inner diameter filled with ReproSil-Pur 120 C18-AQ (Dr Maisch GmbH) mounted to an Easy-nLC 1200 (Thermo Fisher) and sprayed online into an Orbitrap Exploris 480 mass spectrometer (Thermo Fisher). We used a 103-min gradient from 3% to 40% acetonitrile with 0.1% formic acid at a flow of 250 nl/min. The mass spectrometer was operated with a top 20 MS/MS data-dependent acquisition scheme per MS full scan. Mass spectrometry raw data were searched using the Andromeda search engine (Cox et al., 2011) integrated into MaxQuant software suite 1.5.2.8 (Cox and Mann, 2008) using the TriTrypDB-46_TbruceiTREU927_AnnotatedProteins protein database (11,203 entries). For the analysis, carbamidomethylation at cysteine was set as a fixed modification while methionine oxidation and protein N-acetylation were considered as variable modifications. The match between run option was activated.

### Bioinformatics analysis

Contaminants, reverse database hits, protein groups only identified by site, and protein groups with less than two peptides (at least one of them classified as unique) were removed by filtering from the MaxQuant proteinGroups file. Missing values were imputed by shifting a beta distribution obtained from the LFQ intensity values to the limit of quantitation. Further analysis and graphical representation was done in the R framework (Core Team and Others, 2013) incorporating ggplot2 package in-house R scripts (Wickham, 2016).

The mass spectrometry proteomics data have been deposited to the ProteomeXchange Consortium via the PRIDE (Perez-Riverol et al., 2019) partner repository with the dataset identifier PXD019665.

### Ultrastructure expansion microscopy

*T. brucei* cells were processed as indicated above for immunofluorescence. The protocol was adapted after Gambarotto et al. (2019). After the last PBS wash, 150 μl containing 2×10^6^ cells were settled for 20 min at room temperature on poly-D-lysine functionalized coverslips (12 mm, Menzel-Glaser). Coverslips were transferred into 24-well plates filled with a solution of 0.7% formaldehyde (FA, 36.5–38%, Sigma) with 1% acrylamide (AA, 40%, Sigma) in PBS and incubated for 5 h at 37°C. Cells were then prepared for gelation by carefully putting coverslips (cells facing down to the gelling solution) into a 45 μl drop of monomer solution [sodium acrylate (SA, 97–99%, Sigma) 10% (w/w) AA, 0.1% (w/w) N,N′-methylenebisacrylamide (BIS, Sigma) in PBS] supplemented with 0.5% ammonium persulfate (APS) and 0.5% tetramethylethylendiamine (TEMED) on parafilm in a pre-cooled humid chamber. Gelation proceeded for 5 min on ice, and then samples were incubated at 37°C in the dark for 1 h. Coverslips with gels were then transferred into a six well plate filled with denaturation buffer (200 mM SDS, 200 mM NaCl, and 50 mM Tris-HCl in ultrapure water, pH 9) for 15 min at room temperature. Gels were then detached from the coverslips with tweezers and moved into a 1.5-ml Eppendorf centrifuge tube filled with denaturation buffer, and incubated at 95°C for 90 min. After denaturation, gels were placed in beakers filled with deionized water for the first round of expansion. Water was exchanged at least twice every 30 min at room temperature, and then gels were incubated overnight in deionized water. Next day, gels were washed two times 30 min in PBS and subsequently incubated on a shaker (gentle) with primary antibodies anti-polyE (for the polyglutamate chain; AG-25B-0030-C050; Adipogen; anti-PolyE recognizes C-terminally located linear glutamate chains of four and more glutamate residue on α- and β-tubulin), monoclonal mouse-anti-TAC102 antibody (Trikin et al., 2016), rabbit-anti-HA (Sigma) and guinea pig-anti-tubulin (AA345-Gp Expasy Geneva Antibody Facility) at 1:500 diluted in 2% PBS in BSA for 3 h at 37°C. Gels were then washed in phosphate-buffered saline with 0.1% Tween (PBST) three times for 10 min while gently shaking and subsequently incubated with the secondary antibodies DyLight 594 anti-guinea pig IgG (Thermo Fisher cat. SA5-10096), Alexa Fluor 488 goat anti-rabbit IgG (Invitrogen), Alexa Fluor 594 goat anti-mouse IgG (Molecular probes) at 1:500, and DAPI at 1:1000 diluted in 2% BSA in PBS for ∼3 h at 37°C. Gels were then washed in PBST three times for 10 min while gently shaking and finally placed in beakers filled with deionized water for expansion. Water was exchanged at least twice every 30 min before gels were incubated in deionized water overnight. Gel expanded between 3.61× and 3.86× according to SA purity. The cells were imaged using a Leica SP8 STED microscope with 63× objective, *z*-step size of 0.3 µm and zoom factor six.

The expansion factor was determined by comparing the ratio of expanded basal body, kDNA and nucleus to non-expanded basal body, kDNA and nucleus. For unexpanded kDNA and nucleus measurements, *n*=22 cells from immunofluorescence imagery were analyzed. For unexpanded basal body measurements, transmission electron microscopy imagery was used (*n*=12). For measurements on the expanded cells, *n*=22 cells were analyzed. We selected only nearly perfect side-view kDNA for measurement of kDNA length in each cell. The diameter of the nucleus was determined by measuring the widest diameter observed in each cell. The diameter of the basal body was determined by using the plot profile tools of Fiji to plot the Gaussian distribution, then the distance between the first and the last peak of intensity was measured.

### Digitonin fractionations

To analyze biochemical properties of TAP110, we performed digitonin fractionation as described previously (Amodeo et al., 2018). 5×10^6^ cell equivalents of each fraction was used for the western blot analysis. The samples were boiled for 5 min at 95°C in Laemmli buffer for SDS-PAGE.

### Blue native analysis

To detect protein complexes, Blue Native PAGE analysis was performed as described previously (Hoffmann et al., 2018). In brief, 10^8^ cell equivalents for each extract was used for the analysis. Crude mitochondrial fractions (obtained by extraction with 0.025% digitonin) were lysed with 1% digitonin and centrifuged for 15 min at 13,500 ***g***. The supernatant was loaded on a blue native gel, protein complexes were separated by electrophoresis, then the gel was soaked in SDS running buffer and the proteins were transferred onto a PVDF membrane by semi dry western blotting.

### PCR to amplify mini-and maxicircles from gDNA from dyskinetoplastic cells

We used primers (minicircle forward, 5′-TATGGGCGTGCAAAAATACA-3′; minicircle reverse, 5′-CGAAGTACCTCGGACCT-3′; Cox2 forward, 5′-CTAACATACCCACATAAGACAG-3′; Cox2 reverse, 5′-ACACGACTCAATCAAAGCC-3′) designed to amplify whole minicircles and the Cox2 gene on the maxicircles. We did not detect any minicircle DNA in the dyskinetoplastic cells. With wild-type DNA, we obtained the expected product of 767 bp in the maxicircle PCR. For the dyskinetoplastic cells, we obtained a product of ∼1200 bp. We sequenced that PCR product and verified that it is not a product from amplification of maxicircle DNA, but rather a product from amplification of an intergenic region of the nuclear genome. There was a very weak band of ∼800 bp visible as well. However, we were unable to validate this in a repetition of the same PCR (data not shown).

### Flagellar extraction

For flagellar preparation (Ogbadoyi, 2003), we performed the experiment as described previously (Amodeo et al., 2018). For DNase treatments, a second extraction buffer was supplemented with DNaseI (Roche) to a final concentration of 100 μg/ml.

### Phylogenetic analysis of TAP110 and TAC102

Phylogenetic analysis was performed using the Phylo.fr package (Dereeper et al., 2008). Sequences were aligned using the MUSCLE algorithm with standard settings (Edgar, 2004). Phylogenetic tree reconstruction was performed using the PhylML 3.0 algorithm with standard settings (Guindon et al., 2010). Tree visualization and comparison was performed using the Phylo.io tool (Robinson et al., 2016).

### kDNA size measurements in DAPI-stained cells

To measure the change of kDNA networks size upon day 6 of TAP110 depletion, we spread ∼10^6^ uninduced or cells at day 3 post induction onto slides. The experiment was performed as described previously (Amodeo et al., 2018). In brief, the cells on a slide were fixed in cold methanol for 5 min at −20°C. Afterwards, the slides were washed with PBS and then mounted with ProLong^®^ Gold Antifade Mounting Medium containing DAPI. The images were acquired with a 100× oil immersion objective and analyzed using the ImageJ software. We measured the particle size in arbitrary units (a.u.). A cut-off of >0.01 a.u. and ≤1.0 a.u. was made to exclude nuclei from the data set.

For the other quantifications of kDNA phenotypes, we defined the parameters as follows. A cell was defined as a dK1N cell when two TAC102 signals were observed. We previously showed that two TAC signals are associated with dK1N cells (Hoffmann et al., 2018). For small and big kDNA, the kDNA had to be significantly larger than a dividing kDNA or significantly smaller than a regular size kDNA (by eye). Additionally, the cells with smaller or larger than normal kDNA had to have only one TAC102 signal to avoid any confusion with replicating kDNAs.

## Supplementary Material

Supplementary information

Reviewer comments
