# Supplementary material for: Characterization of the novel mitochondrial genome segregation factor TAP110 in Trypanosoma brucei
Source: J Cell Sci. 2021 Mar 8;134(5):jcs254300. doi: 10.1242/jcs.254300 (PMC7970207; doi:10.1242/jcs.254300)
Supplement: Supplementary information [file joces-134-254300-s1.pdf]

TAP110 supplementary information

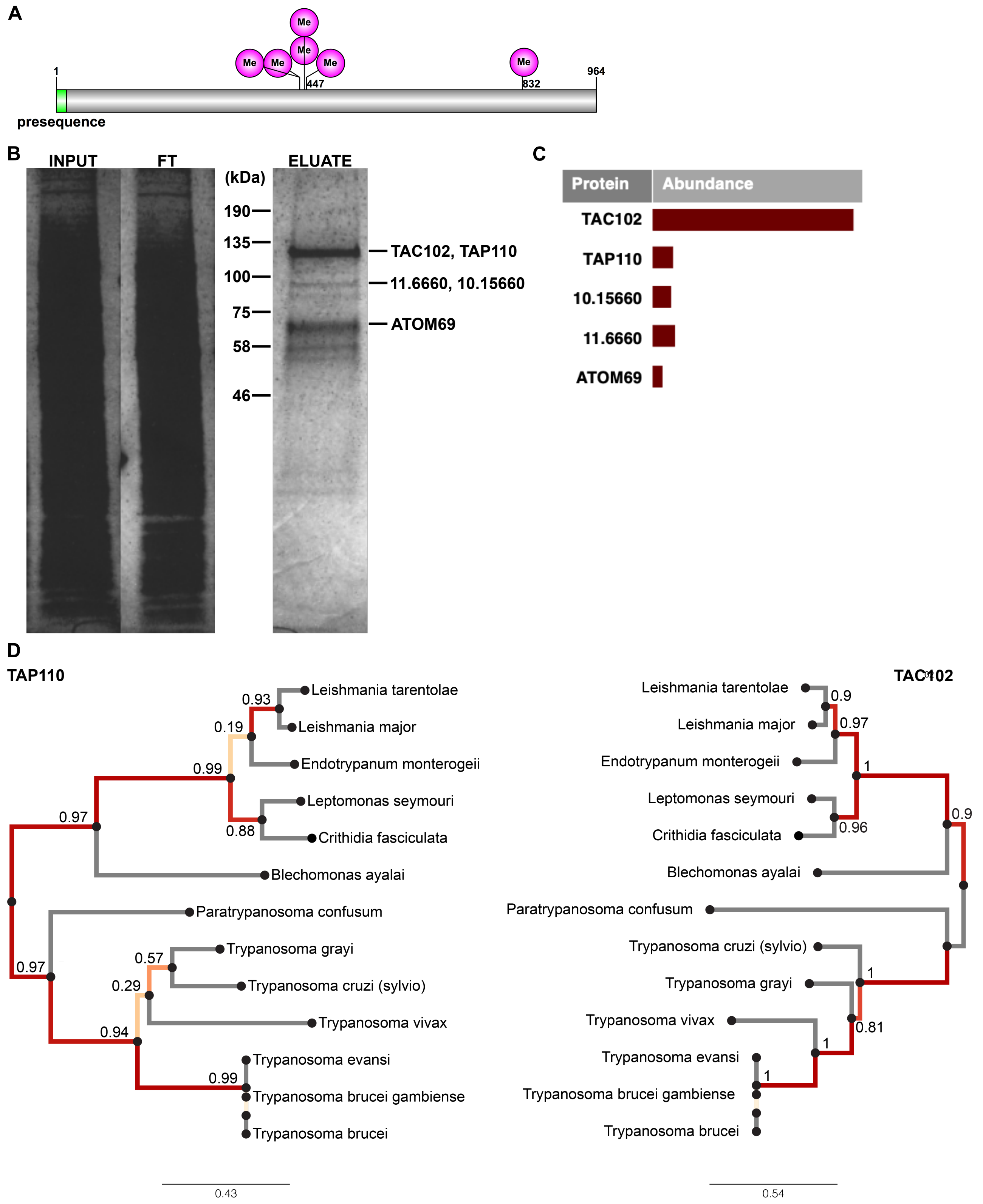

Journal of Cell Science • Supplementary information

Figure S1: Protein domains, interaction partners and phylogeny of the TAC associated protein 110 (TAP110).

A) Illustration of the TAP110 ORF. Highlighted in green, the mitochondrial-targeting sequence; in magenta, the posttranslational modification in form of methylarginine; in light blue, the similarity to the micronuclear linker histone protein of *Tetrahymena thermophila*. **B)** Coomassie gel of a PTP-TAC102 immunoprecipitation (IP) using IgG beads. FT, flow through. **C)** Abundance of TAC102 and its potential interaction partners detected in the eluate of the IP. **D)** A phylogenetic tree showing the conservation of TAC102 and TAP110 among Kinetoplastids. The scale bar indicates the number of amino acid substitutions. Colors indicate support for the branching order at the respective nodes, red (strong support) to grey (weak support and terminal nodes).

Journal of Cell Science • Supplementary information

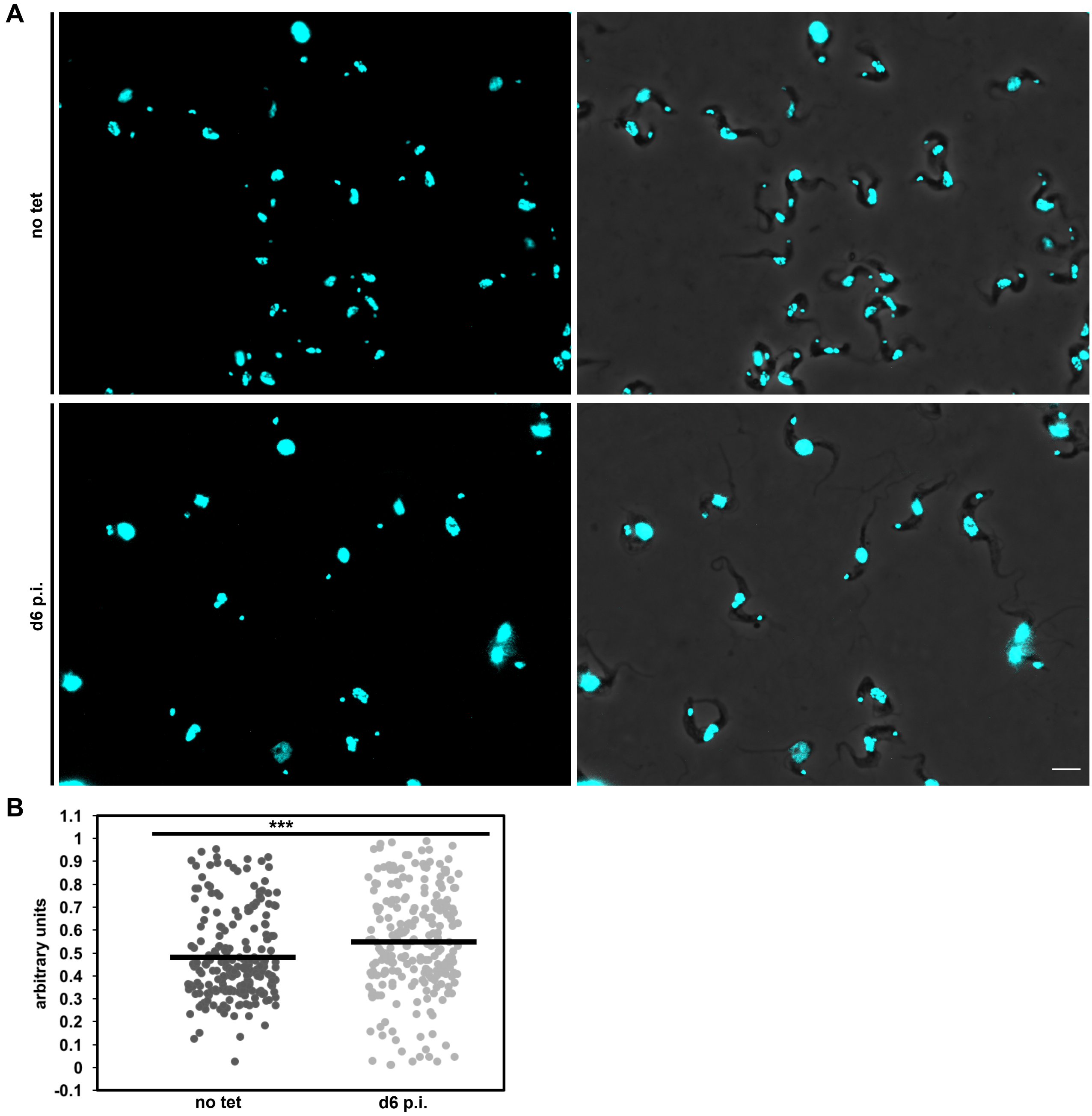

Figure S2: Measurement of kDNA content TAP110 RNAi in BSF cells.

A) Immunofluorescence pictures from non-induced cells (no tet) and cells at day six of the TAP110 RNAi (d6 p.i.). Left side DAPI staining, right side overlay of DAPI with phase contrast. **B**) Size measurements of kDNA DAPI signals from uninduced (no tet; mean = 0.476 arbitrary units) and induced (d6 p.i.; mean = 0.547 arbitrary units) TAP110 RNAi BSF cells (a number of ≥221 areas of kDNAs for each condition were measured). Significance of difference in size was calculated by two-tailed unpaired t- test. *** = $p \leq 0.001$.

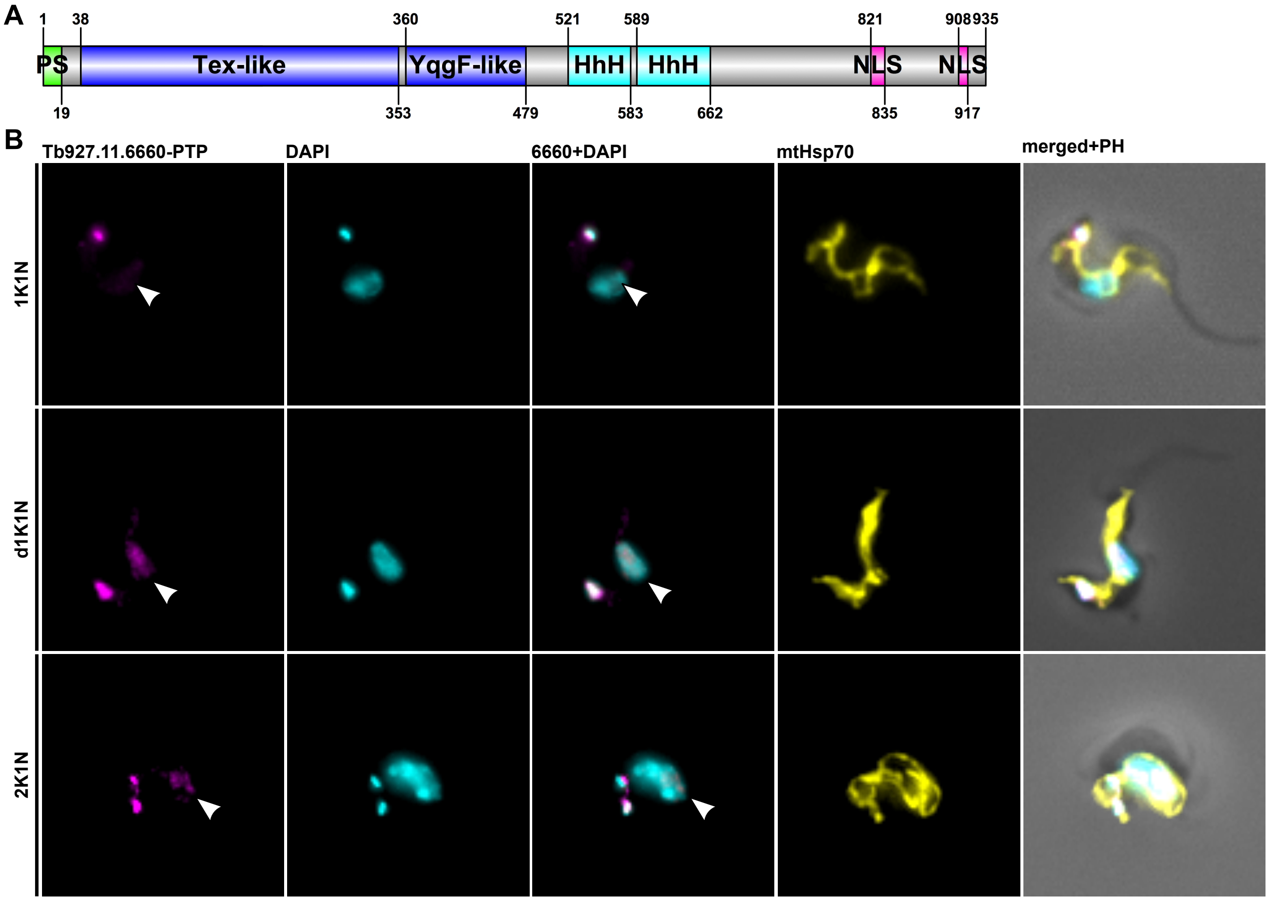

Figure S3: Protein domains and localization of Tb927.11.6660.

A) Illustration of the Tb927.11.6660 ORF. Highlighted in green, the mitochondrial-targeting sequence; in dark blue, the Tex-like and YggF-like domains; in cyan, the helix-hairpin-helix motifs and in magenta, the nuclear localization signals. **B**) Immunofluorescence microscopy images of BSF cells expressing Tb927.11.6660-PTP during different stages of the cell cycle (1K1N, dK1N, 2K1N). Tb927.11.6660-PTP was detected with an anti-Protein A antibody. mtHsp70 with an anti-mtHsp70 antibody and the DNA with DAPI. D, duplicating/duplicated; K, kinetoplast; N, nucleus; PH, phase contrast, 6660, Tb927.11.6660-PTP.

Journal of Cell Science • Supplementary information

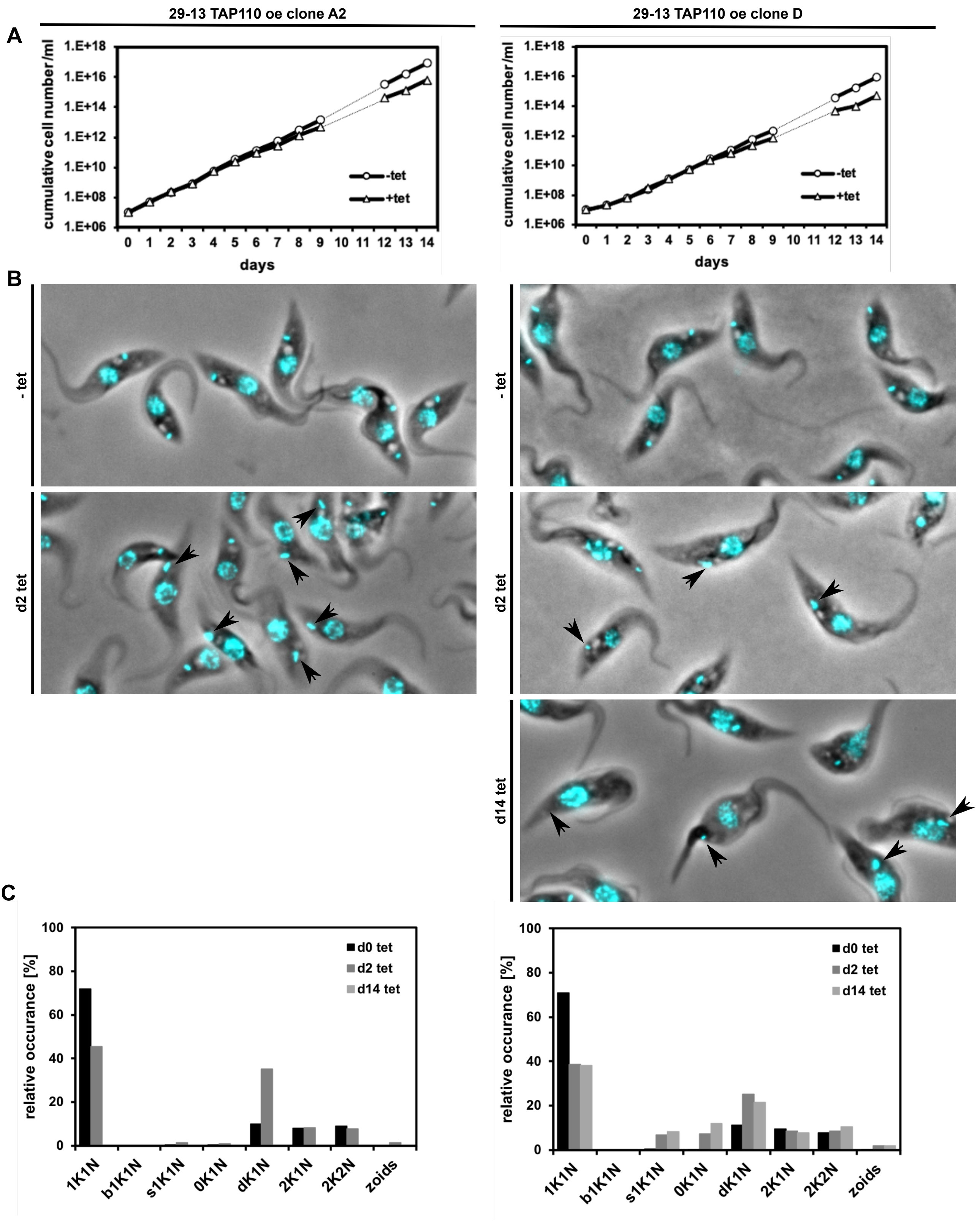

Figure S4: Growth curve and quantification of DAPI stained cells from procyclic form 29-13 tetracycline inducible TAP110 overexpression clone A2 and D.

A) Growth curves. **B)** MeOH fixed and DAPI stained cells. -tet, uninduced cells; d2 tet, cells at day two of the overexpression; d14 cells at day 14 of the overexpression. Arrowheads point to abnormal kDNAs or duplicating/duplicated kDNAs. **C)** Quantification of the relative occurrence of kDNA networks and nuclei from experiment shown in B). >294 cells were counted per condition (d0, d2, d14). b, big; d, duplicating/ duplicated; K, kinetoplast; N, nucleus; s, small; zoids, cells lacking nucleus

Journal of Cell Science • Supplementary information

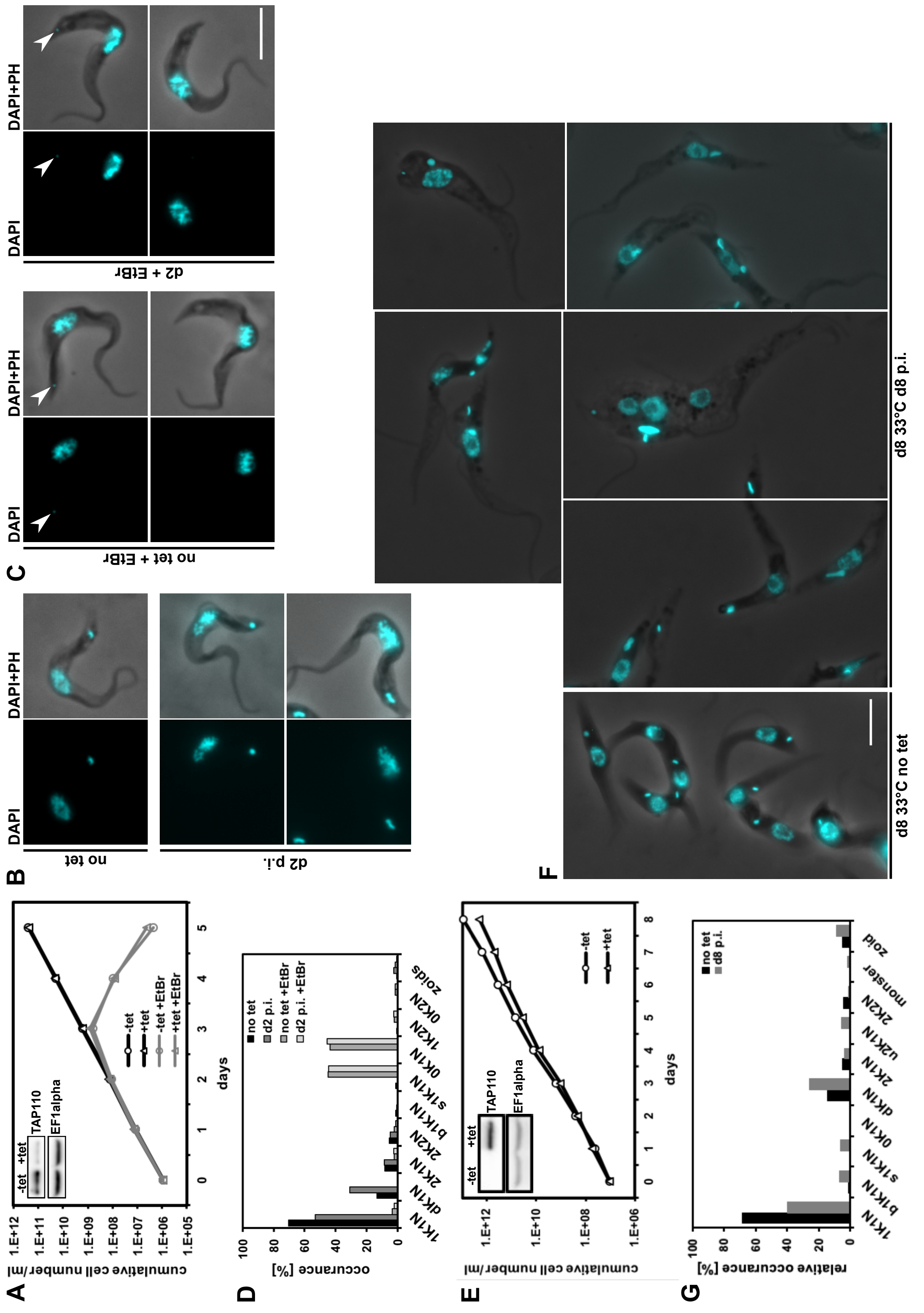
A
B
C
D
E
F
G
cumulative cell number/ml
days
-tet
+tet
-tet +EtBr
+tet +EtBr
TAP110
EF1alpha
no tet
d2 p.i.
DAPI
DAPI+PH
no tet + EtBr
d2 + EtBr
occurance [%]
no tet
d2 p.i.
no tet +EtBr
d2 p.i. +EtBr
1K1N
dK1N
2K1N
2K2N
b1K1N
s1K1N
0K1N
1K2N
0K2N
zoids
d8 33°C no tet
d8 33°C d8 p.i.
relative occurance [%]
d8 p.i.
u2K1N
monster
zoid

Figure S5: Ethidium bromide and heat stress experiment with the TAP110 RNAi BSF and TAP110-HA overexpression PCF cell line.

A) Growth curve of tet inducible BSF TAP110 RNAi cells either grown without or with EtBr. The inset depicts a western blot, showing depletion of TAP110 at day one post induction. EF1alpha serves as a loading control. **B)** Representative fluorescence microscopy images of non-induced cells and cells at day two post induction which were not treated with EtBr. The nucleus and the kDNA were stained with DAPI. **C)** Representative fluorescence microscopy images of non-induced cells and cells at day two post induction which were treated with EtBr. Arrowheads point to small kDNA. **D)** Quantification of the relative occurrence of kDNA discs and nuclei from the experiment shown in C). A number of ≥150 cells per condition were analyzed. **E)** Growth curve of tet inducible TAP110 overexpression cells grown at 33°C. The inset depicts a western blot, showing expression of TAP110-HA at day one post induction. EF1alpha serves as a loading control. **F)** Representative fluorescence microscopy images of non-induced cells and cells at day eight post induction which were both grown for eight days at 33°C. Same staining procedure as in C was used. **G)** Quantification of the relative occurrence of kDNA discs and nuclei from the experiment shown in F). A number of ≥150 cells per condition were analyzed. bK, big kDNA; dK, duplicating kDNA; K, kDNA; N, nucleus; PH, phase contrast; sK, small kDNA; u2K1N, unequal sized kDNAs = missegregated kDNAs. Scale bars: 5 µm.

Journal of Cell Science • Supplementary information

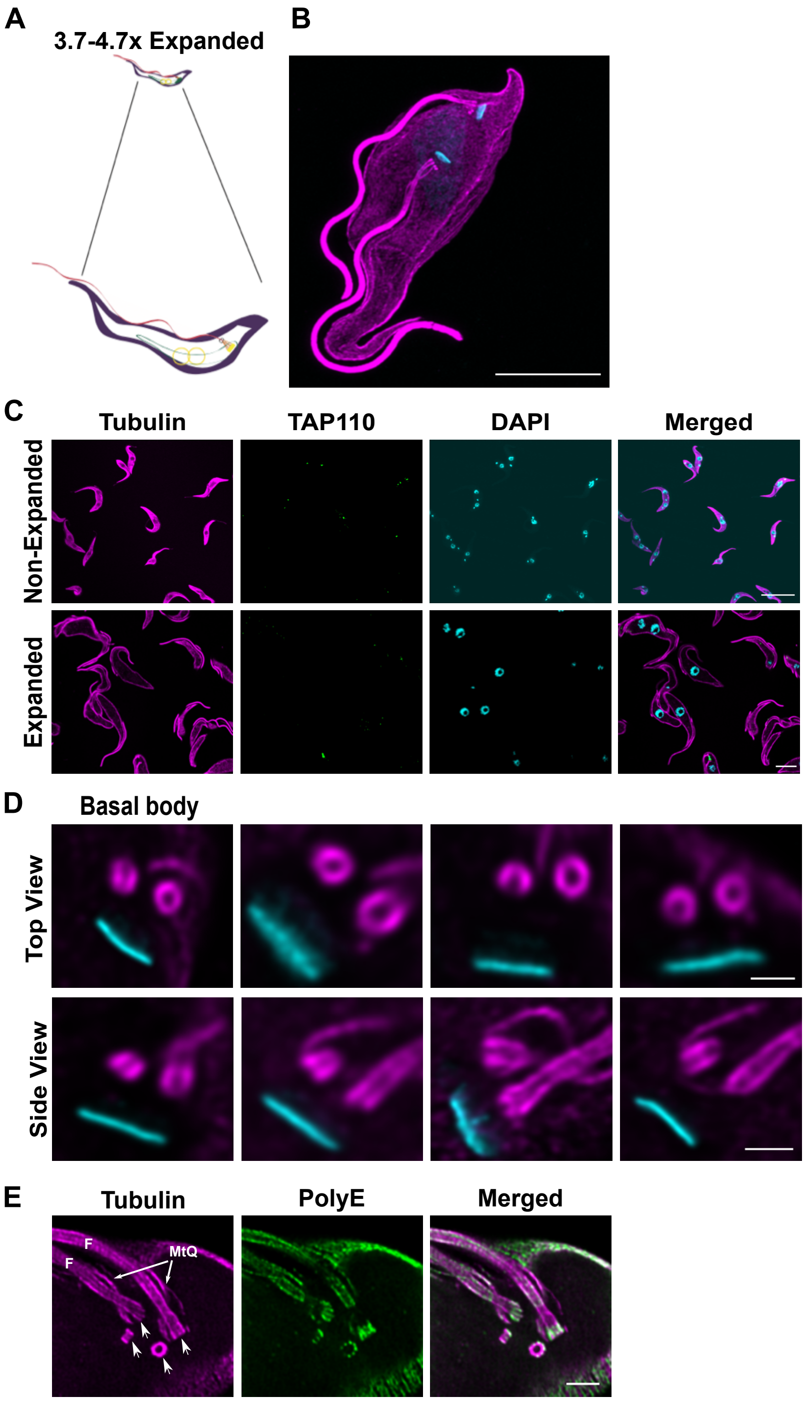

Figure S6: Isotropic expansion of *T. brucei* PCF whole cells and different subcellular structures by U-ExM.

A) Schematic view of the expansion concept in *T. brucei*. **B)** Maximum intensity projection of an expanded PCF cell stained with anti-tubulin (magenta; Alexa Fluor 594) and DAPI (cayan) to illustrate a side view of a cell in a good orientation. Scale bar: 20 µm. **C)** Representative single plane images of expanded and non-expanded PCF cells stained with anti-tubulin (magenta; Alexa Fluor 594), anti-HA for TAP110 (green; Oregon Green 488) and DAPI for the kDNA and the nucleus (cayan). The cells were imaged by confocal microscopy and the imagery was deconvoluted by Huygens professional software. The z plane was selected based on nearly perfect view of the cells, therefore only one cell has TAP110 signal in the given single plane. Scale bar: 20 µm. **D)** Representative confocal images (deconvolved) of *T. brucei* basal bodies and kDNAs

Journal of Cell Science • Supplementary information

(stained as described above) from 2 different experiments. Scale bar: 2 µm. **E)** PCF cells stained for PolyE (green; Alexa Fluor 488) and anti-tubulin (magenta; Alexa Fluor 568) and imaged by confocal microscopy followed by HyVolution. Scale bar: 2 µm. Arrows point to the basal bodies. F, Flagella; MtQ, microtubule quartet

Journal of Cell Science • Supplementary information

Table S1: Proteins changed in abundance upon overexpression of TAP110-HA.

TrypDB Gene ID	protein name	enrichment	p-value	IEP	mt prediction	localization TrypTagDB	localization TriTrypDB
Tb927.11.7590	TAP110	2.15	0.0070	7.98	++	bb	-
Tb927.5.1720	HYP	1.89	0.0673	8.65	+++	mt, kDNA(strong)	-
Tb927.11.6660	Tex-like N-terminal domain / Helix-hairpin-helix motif containing protein	0.54	0.0001	9.85	++	kDNA, nucleolus(weak)	-
Tb927.4.3080	ACR containing / YggU-like protein	-1.52	0.0186	8.45	+++	-	-
Tb927.8.4550	mt ribosomal SSU protein	-1.47	0.0417	10.53	++	-	-
Tb927.10.3880	HYP	-1.35	0.0366	7.00	+	cytoplasm	-
Tb927.5.3870	mt ribosomal LSU protein	-1.05	0.0359	7.03	++	-	-
Tb927.4.2520	SIR2rp3	-1.03	0.0307	6.42	-	mt	-
Tb927.11.9630	Snf7	1.14	0.0130	4.55	-	endocytic	-
Tb927.3.2040	kinesin	1.14	0.0479	5.83	-	hook complex	-
Tb927.8.7440	lipase	1.04	0.0127	7.04	-	endocytic	-
Tb927.11.10120	HYP	0.68	0.0155	10.27	-	-	flagellum
Tb927.11.2070	HYP	-3.09	0.0008	7.96	-	-	-
Tb927.8.3590	HYP	-1.56	0.0484	9.95	-	-	-
Tb927.4.4710	HYP	-0.72	0.0127	4.65	-	cytoplasm, flagellum	flagellum

The enrichment of a protein is displayed as log(2) value. The mt prediction shows likelihood of a protein being mitochondrial. ACR, ADAM cysteine-rich domain (ADAMs family proteins belong to the zinc protease superfamily); HYP, hypothetical protein, conserved; IEP, isoelectric point; mt, mitochondrion/mitochondrial; SIR2rp3, silent information regulator 2 related protein 3 (SIR proteins catalyze deacetylation of histones as well as nonhistone proteins); Snf7, this family of proteins are involved in protein sorting and transport from the endosome to the vacuole/lysosome in eukaryotic cells; Tex, toxin expression; +, mitochondrial prediction by either the Mitocarta data set (Zhang et al., 2010), the mitochondrial importome data set (Peikert et al., 2017) or by Mitoprot (Claros and Vincens, 1996); ++, mitochondrial prediction by two of the three parameters mentioned above; +++, mitochondrial prediction all three parameters mentioned above

Journal of Cell Science • Supplementary information
