# Supplementary material for: Characterization of the novel mitochondrial genome segregation factor TAP110 in Trypanosoma brucei
Source: J Cell Sci. 2021 Mar 8;134(5):jcs254300. doi: 10.1242/jcs.254300 (PMC7970207; doi:10.1242/jcs.254300)
Supplement: Reviewer comments [file joces-134-254300_review_history.pdf]

Characterization of the novel mitochondrial genome segregation factor TAP110 in *Trypanosoma brucei*

Simona Amodeo, Ana Kalichava, Albert Fradera-Sola, Eloïse Bertiaux-Lequoy, Paul Guichard, Falk Butter and Torsten Ochsenreiter
DOI: 10.1242/jcs.254300

Editor: Jennifer Lippincott-Schwartz

Review timeline
Original submission: 11 September 2020
Editorial decision: 26 October 2020
First revision received: 21 December 2020
Accepted: 3 February 2021

Original submission

First decision letter

MS ID#: JOCES/2020/254300

MS TITLE: Characterization of the Novel Mitochondrial Genome Segregation Factor TAP110 in Trypanosoma brucei

AUTHORS: Simona Amodeo, Ana Kalichava, Albert Fradera-Sola, Eloise Bertiaux-Lequoy, Paul Guichard, Falk Butter, and Torsten Ochsenreiter
ARTICLE TYPE: Research Article

We have now reached a decision on the above manuscript.

To see the reviewers' reports and a copy of this decision letter, please go to: https://submit-jcs.biologists.org and click on the 'Manuscripts with Decisions' queue in the Author Area. (Corresponding author only has access to reviews.)

As you will see, the reviewers raise a number of substantial criticisms that prevent me from accepting the paper at this stage. They suggest, however, that a revised version might prove acceptable, if you can address their concerns. If you think that you can deal satisfactorily with the criticisms on revision, I would be pleased to see a revised manuscript. We would then return it to the reviewers.

We are aware that you may be experiencing disruption to the normal running of your lab that makes experimental revisions challenging. If it would be helpful, we encourage you to contact us to discuss your revision in greater detail. Please send us a point-by-point response indicating where you are able to address concerns raised (either experimentally or by changes to the text) and where you will not be able to do so within the normal timeframe of a revision. We will then provide further guidance. Please also note that we are happy to extend revision timeframes as necessary.

Please ensure that you clearly highlight all changes made in the revised manuscript. Please avoid using 'Tracked changes' in Word files as these are lost in PDF conversion.

© 2021. Published by The Company of Biologists under the terms of the Creative Commons Attribution License (https://creativecommons.org/licenses/by/4.0/).

I should be grateful if you would also provide a point-by-point response detailing how you have dealt with the points raised by the reviewers in the 'Response to Reviewers' box. Please attend to all of the reviewers' comments. If you do not agree with any of their criticisms or suggestions please explain clearly why this is so.

Reviewer 1

Advance summary and potential significance to field

Kinetoplastids possess a unique mitochondrial genome architecture and mechanism for mt genome segregation, through linkage to the basal bodies. Recent work from the Ochsenreiter lab and others, has significantly advanced the knowledge about the molecular architecture of TAC. In this paper, Amodeo et al. add to this, by characterising a new TAC protein TAP110, which interacts with TAC102. They show there is co-dependency between TAC102 and TAP110, as assessed in their assembly after kDNA loss. TAP110 is the most kDNA-proximal component that has been found. Removing DNA increases the solubility of TAP110 but whether it binds DNA directly could not yet be determined. The nature of the linkage between the kDNA and the TAC is an important open question and this study is a step closer to answering it.

Comments for the author

This is a well-executed study, with solid data supporting the main claims. There are really only very minor issues the authors may want to clarify or expand upon.

1. The authors say TAP110 and TAC102 “tend to be inherited together” but it is unclear what “phylogenetic comparison was made to show that. The tree shown in supplemental figure 1D, which is provided as evidence would probably look very similar if two functionally unrelated kinetoplastid proteins were analysed. The absence of both proteins in Perkinsela and Bodo saltans was mentioned in the discussion. Is that the basis for the conclusion that they are “inherited together” or is there evidence of absence of both proteins for a wider number of genomes?

2. There is speculation about a link between mitochondrial and nuclear communication based on a change in expression level for Tb927.11.6660, which is reported to have strong similarities to TEX proteins. It is unclear whether the authors suggest that it is the similarity to TEX that would support a potential link between replication/segregation in the two compartments, and if so what specific functions or properties of TEX-like proteins would support this, or whether their speculative link is solely based on the dual localisation.

3. The expansion microscopy certainly provides stunning images of the cytoskeleton. Although “revolutionary” (line 42) is perhaps too strong a word to describe this method, it certainly has potential to facilitate further dissection of cellular architecture. In the methods section, the authors should detail how cells treated for expansion microscopy were imaged. The legend to Fig S6 only says “confocal microscopy” - was the microscopy the same as described for standard immunofluorescence analysis?

4. For quantification of the expansion, basal bodies imaged by TEM were used as reference. How was shrinkage of samples that occurs during TEM sample preparation considered in these calculations?

Line 39, typo: TAP110 (not TA110)
Line 46, what are “derived organelles” of mitochondria?

Reviewer 2

Advance summary and potential significance to field

Mitochondrial DNA replication and maintenance are of broad interest, and the mechanisms behind segregation of the extranuclear genome are just now being uncovered. The tripartite attachment

© 2021. Published by The Company of Biologists under the terms of the Creative Commons Attribution License (https://creativecommons.org/licenses/by/4.0/).

complex (TAC) in trypanosomatids is an elaborate feature designed to segregate the kDNA. In recent years, essential TAC components have been identified and a hierarchy of TAC assembly has been reported. However, a protein that directly connects to the kDNA has not been identified. For this manuscript the authors present data that describes a new TAC associated protein - TAP110.

They authors use RNAi and overexpression (OE) to study the function and TAC hierarchy of TAP110 and identify a protein that increased in abundance in response to TAP110 OE. Of special significance is the application of expansion microscopy to view the nanostructure of the kDNA network. This is beautiful work and an important contribution to their field. The experiments are clear, relevant and the quality of the data appears to be high, but can be strengthened as indicated below. Results generally support their conclusions, but there are several occasions that the authors are over-interpreting the data. The level and quality written English is of a high standard.

The work provides new insights into kinetoplastid specific proteins that maintain the novel kDNA structure. Data presented here are interesting, however, the role of TAP110 has not really been resolved nor has the role for 11.6660. The work is of high enough quality and novelty to be published in J. Cell. Science, but only if the points below are addressed and lends more data for mechanistic insight. Additional comments and questions are listed below.

Comments for the author

1. In the abstract the authors state “depletion as well as overexpression of TAP110 leads to a delay in the separation of the replicated kDNA networks”. This is implying a segregation defect. Also, at the end of the discussion (line 349-350) the authors state they have described a novel kDNA segregation factor. Earlier in the discussion the authors do indicate that this is not a classical segregation protein so why highlight this in the abstract and now how to define the role of this protein. Perhaps the authors could refer to the review by Mensa-Wilmot where kinetoplast division factors are further classified based on trypanosome defects following failure to complete specified steps of the kinetoplast cycle.

Additional experiments are needed to fully evaluate the role of TAP110. The delay in segregation for both RNAi and OE could be a secondary effect. TAP110 could be interacting (perhaps transiently) and this would also explain the delay in the segregation defect. It is of interest that there is a significant impact on the percentage of cells that are listed a dK1N (for both RNAi and OE). The OE data presented indicates that this pattern is seen at later day of OE as well. So then why is there NO impact on the fitness of the cell? Wouldn’t this impact cell cycle progression and thus doubling time?
It almost appears as if the cells have become synchronized.

2. Regarding the RNAi or OE phenotypes – why not perform the heat stress on the BSF RNAi as well? Although this would require a higher temp it would be more comparable. Treating the cells with EtBr is mutagenic. Along this experimental line, why not perform the proteome analysis on the heat stressed PCF cells and compare the two? If their hypothesis is correct that 11.6660 is involved in signaling from the mito -> nucleus then any changes associated with that proteome under stress would be informative.

3. Only two of the figures (4 and 5) include error bars and an indication of biological replicates. Before this body of work can be published the authors need to demonstrate more than just a single biological replicate for their data and include error bars.

4. Point of clarification: measurement for kDNA size is reported as arbitrary units. What were the parameter that allowed distinction among 1K1n, dK1N, s1K1N and b1K1N? Authors mention the cut-off of arbitrary units, but not what those number correlate to.

5. Use of the term “kDNA associated”: do the authors truly mean that the proteins are associated with the kDNA? If so, they have not directly demonstrated this feature of TAP110 or 11.6660. They indeed do localize near the kDNA but this reviewer could not find any evidence for a direct interaction with the kDNA.

© 2021. Published by The Company of Biologists under the terms of the Creative Commons Attribution License (https://creativecommons.org/licenses/by/4.0/).

6. Intriguing suggestion that 11.6660 could be involved in mito->nucleus signaling. The authors should explore the relationship between TAP110 and 11.6660 in more detail since the authors highlight that these two proteins were co-discovered using TAC102 IP and the 11.6660 abundance increased when TAP110 is OE. For example,

7. The experiments with the L262 cell line were a nice way to examine the requirement of kDNA for TAP110 localization. The authors use terminology in Figure 6 such as wild type signal and weak signal.

How were these signals quantified – fluorescence intensity? Is so what defines a weak signal vs WT signal? Also related to these experiments – what happens to the protein abundance for TAP110 and TAC102 during the p197 RNAi?

Minor comments:

8. Page 2, line 37, use of the term kDNA associated is vague.

9. Page 2, line 39: correct to read TAP110

10. Page 2, line 46: for the non-specialist would they be familiar with the idea of derived organelles?

11. Page 2, line 50-51: oxidative phosphorylation chain is non-standard terminology

12. Page 2, line 64-65: provide a more precise definition of the KFZ - proximity to basal body

13. Page 3, lines 90-92: reference for pATOM36 appears to be missing; include the replication factor POLIC also localized near TAC102.

14. Page 7, line 200-201: Please comments on the extra staining spots that are seen in Figure 5A for TAP110.

15. Page 7, line 210: please clarify the meaning of “near non-fixed cells”.

16. Page 7-8, line 244-246: please clarify why there appears to be NO large complex formed in the WT cells even though essential all of the protein is in the pellet following the digitonin treatment. Why would only the DK cell line indicate a complex of larger molecular mass?

17. Page 9, lines 312-316: no data is presented regarding partial solubility of TAC102 – reference for this statement?

Figure and Figure legends:

18. Figure 2, is it possible to enlarge the microscopy images and shrink the western blot. Alternatively can the authors just show the enlargements to see the overlap of signal and present the whole cell in sup data? Also, the authors describe difference in fluorescence intensity of TAC102 during TAP110 RNAi related to figure 2F. Include supp data to show this variation that correlates with their description.

19. Figure 6, Please define others. Was TAP110 ever detected without the presence of TAC102?

20. Figure 7C, for the TAC102 panel what do the two different WT and DK lanes represent? Different amount loaded? Different clones?

21. Figure S1A, legend indicates a light blue region similar to tetrahymena – not included in the figure.

22. Figure S1D, what do the different colored lines represent? Use of larger font to discriminate TAP110 vs TAC102 would be helpful

© 2021. Published by The Company of Biologists under the terms of the Creative Commons Attribution License (https://creativecommons.org/licenses/by/4.0/).

23. Figure S2, are the uninduced and induced panels the same magnification? IN general the uninduced cell look smaller – is overall larger cell morphology part of the TAP110 RNAi phenotype?

24. Figure S3, It is extremely hard to view the pattern for 11.6660 in the overlay with the hsp70 marker. What is the associated localization in 2K2N cells? Why not use U-ExM for this interesting protein as well?

25. Figure S4A, error in the X axis for both clones presented.

26. Figure S5C, what happened to all of the TAP110 signal after expansion? Perhaps indicate how much signal is lost upon expansion? It is hard to evaluate from the small images that are provided.

27. Figure 5E legend, please define PolyE (maybe methods?)

First revision

Author response to reviewers' comments

We would like to thank the reviewers for the constructive criticism. We have addressed each of the points of the reviewers and especially improved the statistical analysis by adding technical or biological replicates mounting to a total of almost 2000 additional cells that were analyzed for the RNAi, RNAi recovery and flagellar extraction experiments.

It also came to our attention that we had accidentally added a wrong graph visualizing the expansion factor comparison in Figure 5D. We have now corrected it with the graph that is fitting the numbers in the text. The expansion factor graph that was wrongly included was from a preliminary analysis of the basal body diameter using less accurate measurements. We apologize for this mistake.

Reviewer 1 Advance Summary and Potential Significance to Field:
Kinetoplastids possess a unique mitochondrial genome architecture and mechanism for mt genome segregation, through linkage to the basal bodies. Recent work from the Ochsenreiter lab and others, has significantly advanced the knowledge about the molecular architecture of TAC. In this paper, Amodeo et al. add to this, by characterising a new TAC protein TAP110, which interacts with TAC102. They show there is co-dependency between TAC102 and TAP110, as assessed in their assembly after kDNA loss. TAP110 is the most kDNA- proximal component that has been found. Removing DNA increases the solubility of TAP110 but whether it binds DNA directly could not yet be determined. The nature of the linkage between the kDNA and the TAC is an important open question and this study is a step closer to answering it.

Reviewer 1 Comments for the Author:
This is a well-executed study, with solid data supporting the main claims. There are really only very minor issues the authors may want to clarify or expand upon.

1. The authors say TAP110 and TAC102 “tend to be inherited together” but it is unclear what “phylogenetic comparison was made to show that. The tree shown in supplemental figure 1D, which is provided as evidence, would probably look very similar if two functionally unrelated kinetoplastid proteins were analysed. The absence of both proteins in Perkinsela and Bodo saltans was mentioned in the discussion. Is that the basis for the conclusion that they are “inherited together” or is there evidence of absence of both proteins for a wider number of genomes?

The reviewer is correct, also many other proteins with a similar evolutionary history would show this type of phylogenetic reconstruction. In fact, the point of the analysis is to show that although there is only a “weak” phenotype upon loss/overexpression of TAP110 it was maintained the extant Kinetoplastids (of which we have sequence information) with the exception of Perkinsela and Bodo saltans. The lack of the protein in Perkinsela (where all TAC proteins are missing) is a

© 2021. Published by The Company of Biologists under the terms of the Creative Commons Attribution License (https://creativecommons.org/licenses/by/4.0/).

further indication of functional relevance. We also elaborate on the interesting finding of TAC102 and TAP110 missing in Bodo saltans, that has a very different kDNA arrangement and thus likely requires a very different TAC/kDNA interface.
In order to accommodate the reviewers comments we changed the first sentence to: "A phylogenetic analysis shows that TAC102 and TAP110 share a common evolutionary history and similar to other TAC components TAP110 is not found in Perkinsela." (line 110+111)

2. There is speculation about a link between mitochondrial and nuclear communication based on a change in expression level for Tb927.11.6660, which is reported to have strong similarities to TEX proteins. It is unclear whether the authors suggest that it is the similarity to TEX that would support a potential link between replication/segregation in the two compartments, and if so what specific functions or properties of TEX-like proteins would support this, or whether their speculative link is solely based on the dual localisation.

We speculate on the link between TAP110 and 6660 and on the potential mainly based on the dual localization (colocalization of TAP110 and 660 at the kDNA, and localization of 6660 in the nucleus), the increased abundance of the 6660 during TAP110 overexpression as well as the fact that 6660 was previously found to interact with TAC102, a close interactor of TAP110 (this we describe in the discussion). Tex domain proteins are mostly found in bacteria where they are involved in gene expression regulation, but a similar domain is also found in the eukaryotic Spt6 protein that is involved in transcription and/or as a nucleosome chaperone and thus in nuclear functions. Overall, too little is known about these proteins for more detailed conclusions, but it is of course tempting to speculate that these two proteins might provide a link between the two genomes. As clearly stated, this is a speculation, but we think it a very interesting angle that we will follow up in future.

3. The expansion microscopy certainly provides stunning images of the cytoskeleton. Although "revolutionary" (line 42) is perhaps too strong a word to describe this method, it certainly has potential to facilitate further dissection of cellular architecture. In the methods section, the authors should detail how cells treated for expansion microscopy were imaged. The legend to Fig S6 only says "confocal microscopy" - was the microscopy the same as described for standard immunofluorescence analysis?

The term revolutionary was removed (line 42).
The expansion microscopy was not the same as described for standard immunofluorescence analysis. We added following sentence to the M&M U-ExM: "The cells were imaged using Leica SP8 STED microscope with 63x objective. Z- step size 0.3µm and zoom factor six." (line 499+500) Furthermore, we also added the term standard to the immunofluorescence analysis title (line 399) to prevent confusion.

4. For quantification of the expansion, basal bodies imaged by TEM were used as reference. How was shrinkage of samples that occurs during TEM sample preparation considered in these calculations?

We did not consider the shrinkage factor, since for the imagery that we used for comparison, we did not determine this factor.
In the results section we added the following statement: "…potential shrinkage of thin section electron microscopy imagery was not compensated for…" (line 213+214).
We would like to point out that in the classical publications on Ultrastructure expansion microscopy the expansion factor is calculated based on the expansion of the actual gel. We have gone quite some extra mile in order to show that the expansion is isotropic and actually almost equal for very different structures like the nucleus and the basal body. Of course, we are also lucky with our model system that has an overall polarized body shape such that it would become immediately evident if the expansion would not be equal in all three dimensions (isotropic).

Line 39, typo: TAP110 (not TA110)

We changed that.

Line 46, what are "derived organelles" of mitochondria?

© 2021. Published by The Company of Biologists under the terms of the Creative Commons Attribution License (https://creativecommons.org/licenses/by/4.0/).

Derived organelles are for example mitosomes. In order to avoid confusion, we have shortened the sentence to: "Mitochondrial organelles are a defining feature of eukaryotic cells." (line 46)

Reviewer 2 Advance Summary and Potential Significance to Field:
Mitochondrial DNA replication and maintenance are of broad interest, and the mechanisms behind segregation of the extranuclear genome are just now being uncovered. The tripartite attachment complex (TAC) in trypanosomatids is an elaborate feature designed to segregate the kDNA. In recent years, essential TAC components have been identified and a hierarchy of TAC assembly has been reported. However, a protein that directly connects to the kDNA has not been identified. For this manuscript the authors present data that describes a new TAC associated protein - TAP110.

They authors use RNAi and overexpression (OE) to study the function and TAC hierarchy of TAP110 and identify a protein that increased in abundance in response to TAP110 OE. Of special significance is the application of expansion microscopy to view the nanostructure of the kDNA network. This is beautiful work and an important contribution to their field. The experiments are clear, relevant and the quality of the data appears to be high, but can be strengthened as indicated below. Results generally support their conclusions, but there are several occasions that the authors are over-interpreting the data. The level and quality written English is of a high standard.

The work provides new insights into kinetoplastid specific proteins that maintain the novel kDNA structure. Data presented here are interesting, however, the role of TAP110 has not really been resolved nor has the role for 11.6660. The work is of high enough quality and novelty to be published in J. Cell. Science, but only if the points below are addressed and lends more data for mechanistic insight. Additional comments and questions are listed below.

Reviewer 2 Comments for the Author:

1. In the abstract the authors state "depletion as well as overexpression of TAP110 leads to a delay in the separation of the replicated kDNA networks". This is implying a segregation defect. Also, at the end of the discussion (line 349-350) the authors state they have described a novel kDNA segregation factor. Earlier in the discussion the authors do indicate that this is not a classical segregation protein so why highlight this in the abstract and now how to define the role of this protein. Perhaps the authors could refer to the review by Mensa- Wilmot where kinetoplast division factors are further classified based on trypanosome defects following failure to complete specified steps of the kinetoplast cycle.

TAP110 loss and overexpression indeed lead to an increase of non-segregated kDNA networks. From this, its localization, and interaction with a well characterized TAC component we conclude that the protein is involved in the segregation process. When we compare TAP110 to TAC102, a well described factor of the segregation machinery, we find that TAP110 shows different properties than the "classic" TAC components. Hence, we named it a TAC associated protein. We are not sure as to what the reviewer refers in the Mensa Wilmot review and how that relates to TAP110?

Additional experiments are needed to fully evaluate the role of TAP110. The delay in segregation for both RNAi and OE could be a secondary effect. TAP110 could be interacting (perhaps transiently) and this would also explain the delay in the segregation defect. It is of interest that there is a significant impact on the percentage of cells that are listed a dK1N (for both RNAi and OE). The OE data presented indicates that this pattern is seen at later day of OE as well. So then why is there NO impact on the fitness of the cell? Wouldn't this impact cell cycle progression and thus doubling time? It almost appears as if the cells have become synchronized.

The reviewer is correct that while the overexpression as well as the depletion phenotypes are clearly detectable, they only seem to have a minor effect on cell growth in culture. Our explanation is that the cell cycle overall is mostly independent of the kDNA subcycle, and leads to what the reviewer refers to as synchronization of the cells. Similarly, although there might be

© 2021. Published by The Company of Biologists under the terms of the Creative Commons Attribution License (https://creativecommons.org/licenses/by/4.0/).

integration of the nuclear and mitochondrial genome replication cycle it is clear that akinetoplastic cells for example grow and divide just fine as long as they have acquired compensatory mutations in the ATPase complex. There could of course be a "secondary" effect, however the localization and proteomics studies of this protein clearly indicate a role in the TAC/kDNA. As always there are more experiments that could be performed, but at this stage we do not see how a stronger phenotype would further our understanding of the function of this protein.

2. Regarding the RNAi or OE phenotypes – why not perform the heat stress on the BSF RNAi as well? Although this would require a higher temp it would be more comparable. Treating the cells with EtBr is mutagenic. Along this experimental line, why not perform the proteome analysis on the heat stressed PCF cells and compare thetwo? If their hypothesis is correct that 11.6660 is involved in signaling from the mito -> nucleus then any changes associated with that proteome under stress would be informative.

We used a more general stress (heat) and a stress more specifically targeting the kDNA and its replication (ethidium bromide). Since there is no evidence that kDNA replication or segregation would be different between bloodstream form and procyclic form trypanosomes we do not think repeating the experiments of heat stress in the BSF and ethidium bromide in PCF would add any more information. Our lab is quite experienced in proteomics studies and analyzing the overall proteome upon heat stress is a major experiment that would very likely lead to many new protein candidates which in turn would have to be verified in order to provide any useful insight. This is clearly past the objective of this manuscript, although certainly an interesting experiment to be done.

3. Only two of the figures (4 and 5) include error bars and an indication of biological replicates. Before this body of work can be published the authors need to demonstrate more than just a single biological replicate for their data and include error bars.

We have now performed triplicates for all the quantifications seen in figures 2, 3 6 and 8 and included error bars in the graphs where relevant. Error bars were not included for all time points of the overexpression, since the relevant change occurs on day two post induction, which is also the timepoint when the proteomics samples were taken. The biological replicates two and three for the overexpression are shown in more detail in Figure S4.

4. Point of clarification: measurement for kDNA size is reported as arbitrary units. What were the parameter that allowed distinction among 1K1n, dK1N, s1K1N and b1K1N? Authors mention the cut-off of arbitrary units, but not what those number correlate to.

For the kDNA size measurements of figure S2 we analyzed the images using ImageJ. The kDNA size analysis was performed on binarized 8-bit format images. Particle size was measured in arbitrary units (a.u.) which corresponds to the area. We included kDNA particles of >0.01 a.u. and ≤1.0 a.u. in the analysis, as particles ≤0.01 were seen to be background and particles >1.0 were seen as nuclear genomes. We did not distinguish between 1K and dK and sK and bK in this experiment. For the other experiments we did the following: "A cell was defined as a dK1N cell when two TAC102 signals were observed. We previously showed that two TAC signals are associated with dK1N cells (Hoffmann et al. 2018). For small and big kDNA, the kDNA had to be significantly larger than a dividing kDNA or significantly smaller than a regular size kDNA (by eye). Additionally the cells with smaller or larger than normal kDNA had to have only one TAC102 signal to avoid any confusion with replicating kDNAs." (line 545-550)
We added this information to the materials and methods section.

5. Use of the term "kDNA associated": do the authors truly mean that the proteins are associated with the kDNA? If so, they have not directly demonstrated this feature of TAP110 or 11.6660. They indeed do localize near the kDNA but this reviewer could not find any evidence for a direct interaction with the kDNA.

We use the term associated in the sense of linked to or correlated with. We do not claim any direct interactions with the kDNA. We changed the sentence in question to: "....one was a kDNA linked protein,..." (line 346)

© 2021. Published by The Company of Biologists under the terms of the Creative Commons Attribution License (https://creativecommons.org/licenses/by/4.0/).

6. Intriguing suggestion that 11.6660 could be involved in mito->nucleus signaling. The authors should explore the relationship between TAP110 and 11.6660 in more detail since the authors highlight that these two proteins were co-discovered using TAC102 IP and the 11.6660 abundance increased when TAP110 is OE. **For example,**

Indeed, this is a very exciting finding. We are currently evaluating the role of 11.6660 in retrograde signaling but this is a complete story in its own right and will be published elsewhere.

7. The experiments with the L262 cell line were a nice way to examine the requirement of kDNA for TAP110 localization. The authors use terminology in Figure 6 such as wild type signal and weak signal.
How were these signals quantified – fluorescence intensity? Is so what defines a weak signal vs WT signal? Also related to these experiments – what happens to the protein abundance for TAP110 and TAC102 during the p197 RNAi?

We did these measurements by eye and not signal intensity measurements. We used the basal body signal as reference for the correct position and the wild type signal intensity (as the basal body is not affected by p197 RNAi, as previously shown in Hoffmann et al. 2018. Same intensity as the basal body = wild type, weaker than the basal body = weak.

We furthermore quantified the protein abundance of TAC102 and TAP110-PTP (Figure 6F, G), and could confirm our previous observation that TAC102 protein abundance does not change much during and after p197 depletion (Hoffmann et al. 2018).

Minor comments:

8. Page 2, line 37, use of the term kDNA associated is vague.

The term describes the association, linkage with, colocalization with the kDNA. It is a term commonly used in the annotation of proteins. We would like to keep this here since it is used in many publications.

9. Page 2, line 39: correct to read TAP110

We changed that.

10. Page 2, line 46: for the non-specialist would they be familiar with the idea of derived organelles?

Derived organelles are for example mitosomes. In order to avoid confusion, we have shortened the sentence to: "Mitochondrial organelles are a defining feature of eukaryotic cells." (line 46)

11. Page 2, line 50-51: oxidative phosphorylation chain is non-standard terminology

We changed to: "… mostly belonging to respiratory chain complexes." (line 50).

12. Page 2, line 64-65: provide a more precise definition of the KFZ - proximity to basal body

The KFZ was defined by Drew and Englund as: "the zone between the flagellar face of the disk and the mitochondrial membrane" (Drew and Englund, 2001). *We included the reference and deleted our explanation (line 64).*

13. Page 3, lines 90-92: reference for pATOM36 appears to be missing; include the replication factor POLIC also localized near TAC102.

We included the reference for pATOM36 and the recent discovery of Pol IC as a TAC associated protein and added/changed the following in the manuscript: "Furthermore, there are a number of proteins including TbTBCCD1, pATOM36, alpha-KDE2, AEP1, Pol IC that are in/associated with the TAC and have additional functions in the cell (André et al., 2013; Käser et al., 2016; Miller et al.,

© 2021. Published by The Company of Biologists under the terms of the Creative Commons Attribution License (https://creativecommons.org/licenses/by/4.0/).

2020; Ochsenreiter et al., 2008; Sykes and Hajduk, 2013). The mitochondrial Polymerase IC and the minicircle replication factor MiRF172 furthermore provide evidence for the physical interaction of the replication machinery with the TAC (Amodeo et al., 2018; Miller et al., 2020). (line 90-94)

14. Page 7, line 200-201: Please comments on the extra staining spots that are seen in Figure 5A for TAP110.

These extra staining spots are background signal. We have added the following statement to the results section (line 217-219):"The extra staining seen for TAP110 in the overview image of the expanded cell (Figure 5A), is non-specific staining that occurs with some antibodies at the intersection between the cell and the slide and is visualized due to the maximum intensity projection."

15. Page 7, line 210: please clarify the meaning of "near non-fixed cells".

We have removed the term near-non fixed in order to avoid confusion (line 216).
For explanation: the expansion microscopy protocol includes a step with the fixative paraformaldehyde, however this largely links the gel matrix to the proteins rather than fixing the protein components to each other as a regular fixation protocol would do, hence near-non fixed.

16. Page 7-8, line 244-246: please clarify why there appears to be NO large complex formed in the WT cells even though essential all of the protein is in the pellet following the digitonin treatment. Why would only the DK cell line indicate a complex of larger molecular mass?

This is the case because TAP110 is insoluble in kDNA containing wild type cells. See also Figure 7A.

17. Page 9, lines 312-316: no data is presented regarding partial solubility of TAC102 – reference for this statement?

We have added the references in the text: (Hoffmann et al., 2018; Trikin et al., 2016) *(line 326+327)*

Figure and Figure legends:

18. Figure 2, is it possible to enlarge the microscopy images and shrink the western blot. Alternatively can the authors just show the enlargements to see the overlap of signal and present the whole cell in sup data? Also, the authors describe difference in fluorescence intensity of TAC102 during TAP110 RNAi related to figure 2F. Include supp data to show this variation that correlates with their description.

We have shrunk the western blot and added a zoom-in of the merged images to better see the overlap of signals. Further we have added another representative example of a cell with a weak TAC102 signal (Figure 2C).

19. Figure 6, Please define others. Was TAP110 ever detected without the presence of TAC102?

There are no others mentioned in Figure 6 that we could define. In figure 8 we have now defined the term others (line 685-687).
We did almost never detect TAP110 without TAC102 (in the recovery experiments). This observation supports the idea of an interaction between these two proteins, such as the IP does (Figure S1).

20. Figure 7C, for the TAC102 panel what do the two different WT and DK lanes represent? Different amount loaded? Different clones?

One is the TAP110-tagged cell line, one is the TAC40-tagged cell line. To prevent confusion, we have added the following sentence: "On the blot probed for TAC102 we loaded WT and DK from both cell lines, the TAP110- PTP (left) and the TAC40-HA (right)." (line 670-672).

© 2021. Published by The Company of Biologists under the terms of the Creative Commons Attribution License (https://creativecommons.org/licenses/by/4.0/).

21. Figure S1A, legend indicates a light blue region similar to tetrahymena – not included in the figure.

This text was deleted, as this similarity could not be confirmed when using different blast tools.

22. Figure S1D, what do the different colored lines represent? Use of larger font to discriminate TAP110 vs TAC102 would be helpful

We added a sentence to the figure legend explaining the colors and increased the font size for the labels TAP110 and TAC102 in the phylogenetic tree.
Sentence added: "Colors indicate support for the branching order at the respective nodes, red (strong support) to grey (weak support and terminal nodes)." (supplementary file)

23. Figure S2, are the uninduced and induced panels the same magnification? IN general the uninduced cell look smaller – is overall larger cell morphology part of the TAP110 RNAi phenotype?

Indeed, the microscopic images are of the same magnification in the uninduced and induced panel. Although there is the appearance of increased cell size, the cells did not generally increase in size, rather the number of cells that have replicated their kDNA (but not segregated it) and thus are also in nuclear S-phase (the nucleus is enlarged) has increased. These cells are larger than the average 1K1N wild type cell since they are already further in the cell cycle when cell body growth has started (also refer to Figure 4d in (Jakob et al., 2016)*).*

24. Figure S3, It is extremely hard to view the pattern for 11.6660 in the overlay with the hsp70 marker. What is the associated localization in 2K2N cells? Why not use U-ExM for this interesting protein as well?

We have added an overlay of 6660 and DAPI only for better visibility. We are currently evaluating the role of 6660 and will present this in a different manuscript.

25. Figure S4A, error in the X axis for both clones presented.

We have corrected that.

26. Figure S5C, what happened to all of the TAP110 signal after expansion? Perhaps indicate how much signal is lost upon expansion? It is hard to evaluate from the small images that are provided.

Expansion microscopy increases the actual size of the cell, which means imaging expanded cells will be different in terms of signal distribution when compared to regular microscopy. Furthermore, the image of the expanded cell is from a single stack, whereas the non-expanded cells are maximum intensity projections. We have added this to the figure legend.

27. Figure 5E legend, please define PolyE (maybe methods?)

PolyE is the anti-Polyglutamate chain (polyE) antibody. It is a polyclonal antibody that recognizes C-terminal glutamate chains on alpha- and beta-tubulin.
We added a sentence of clarification to the M&M (line 489+490).

References:

Amodeo, S., Jakob, M. and Ochsenreiter, T. (2018). Characterization of the novel mitochondrial genome replication factor MiRF172 in Trypanosoma brucei. *J. Cell Sci.* **131**, jcs211730.
Drew, M. E. and Englund, P. T. (2001). Intramitochondrial location and dynamics of Crithidia fasciculata kinetoplast minicircle replication intermediates. *J. Cell Biol.* **153**, 735–743.
Hoffmann, A., Käser, S., Jakob, M., Amodeo, S., Peitsch, C., Týc, J., Vaughan, S., Zuber, B., Schneider, A. and Ochsenreiter, T. (2018). Molecular model of the mitochondrial genome segregation machinery in Trypanosoma brucei. *Proc. Natl. Acad. Sci. U. S. A.* **115**,.
Jakob, M., Hoffmann, A., Amodeo, S., Peitsch, C., Zuber, B. and Ochsenreiter, T. (2016). Mitochondrial growth during the cell cycle of Trypanosoma brucei bloodstream forms. *Sci. Rep.* **6**, 36565.

© 2021. Published by The Company of Biologists under the terms of the Creative Commons Attribution License (https://creativecommons.org/licenses/by/4.0/).

Miller, J. C., Delzell, S. B., Concepción-Acevedo, J., Boucher, M. J. and Klingbeil, M. M. (2020). A DNA polymerization-independent role for mitochondrial DNA polymerase IC in African trypanosomes. *J. Cell Sci.* jcs.233072.
Trikin, R., Doiron, N., Hoffmann, A., Haenni, B., Jakob, M., Schnaufer, A., Schimanski, B., Zuber, B. and Ochsenreiter, T. (2016). TAC102 Is a Novel Component of the Mitochondrial Genome Segregation Machinery in Trypanosomes. *PLoS Pathog.* **12**, 1–27.

Second decision letter

MS ID#: JOCES/2020/254300

MS TITLE: Characterization of the Novel Mitochondrial Genome Segregation Factor TAP110 in Trypanosoma brucei

AUTHORS: Simona Amodeo, Ana Kalichava, Albert Fradera-Sola, Eloise Bertiaux-Lequoy, Paul Guichard, Falk Butter, and Torsten Ochsenreiter
ARTICLE TYPE: Research Article

I am happy to tell you that your manuscript has been accepted for publication in Journal of Cell Science, pending standard ethics checks.

Reviewer 1

Advance summary and potential significance to field

Amodeo et al. characterise a new protein, TAP110, associated with the unique mitochondrial genome segregation machinery of trypanosomes, the tripartite attachment complex. In this paper they show TAP110 interacts with the previously characterised protein TAC102. TAP110 is the most kDNA-proximal component that has been found and could be directly involved in DNA binding.

Comments for the author

The authors' response manuscript revisions addressed the comments raised in the first review.

Reviewer 2

Advance summary and potential significance to field

Mitochondrial DNA replication and maintenance are of broad interest due to chronic human disease associated with defects. The mechanisms behind segregation of the mtDNA nucleoids are just now being uncovered. The tripartite attachment complex (TAC) in trypanosomatids is an elaborate feature designed to segregate their unique kDNA. In recent years, the authors (and others) identified essential TAC components and determined a hierarchy of TAC assembly. The authors present data that describes a new TAC associated protein - TAP110. The application of expansion microscopy to view the nanostructure of the kDNA network and associated components is of particular significance for the field.

Comments for the author

The authors addressed numerous comments by adding quantitative data and accompanying statistical analyses, clarified points of inquiry from both reviewers by adding clarifying text to the manuscript, added content to the methods section regarding Expansion Microscopy, enhanced Figure 2C with an additional representative example and a zoomed merge image for each example,

© 2021. Published by The Company of Biologists under the terms of the Creative Commons Attribution License (https://creativecommons.org/licenses/by/4.0/).

updated the graph in Figure 5, updated figures and legends in supplemental info and corrected text where needed.

This work is really quite beautiful and an important contribution to their field setting a new standard for analyzing the trypanosome kDNA network. The experiments are clear, relevant and the quality of the data is high.

© 2021. Published by The Company of Biologists under the terms of the Creative Commons Attribution License (https://creativecommons.org/licenses/by/4.0/).
